# Stability Studies of UV Laser Irradiated Promethazine and Thioridazine after Exposure to Hypergravity Conditions

**DOI:** 10.3390/molecules27051728

**Published:** 2022-03-07

**Authors:** Ágota Simon, Tatiana Tozar, Adriana Smarandache, Mihai Boni, Alexandru Stoicu, Alan Dowson, Jack J. W. A. van Loon, Mihail Lucian Pascu

**Affiliations:** 1 National Institute for Laser, Plasma and Radiation Physics (INFLPR), Laser Department, Atomiștilor 409, 077125 Măgurele, Ilfov, Romania; tatiana.alexandru@inflpr.ro (T.T.); adriana.smarandache@inflpr.ro (A.S.); mihai.boni@inflpr.ro (M.B.); alexstoicu@gmail.com (A.S.); 2 Faculty of Physics, University of Bucharest, Atomiștilor 405, 077125 Măgurele, Ilfov, Romania; 3 European Space Agency (ESA), European Space Research and Technology Centre (ESTEC), TEC-MMG, Keplerlaan 1, 2201 AZ Noordwijk, The Netherlands; alan.dowson@esa.int (A.D.); j.vanloon@amsterdamumc.nl (J.J.W.A.v.L.); 4 Dutch Experiment Support Center (DESC), Department of Oral and Maxillofacial Surgery/Oral Pathology, Amsterdam Bone Center (ABC), Amsterdam UMC Location VU University Medical Center (VUmc) & Academic Centre for Dentistry Amsterdam (ACTA), Gustav Mahlerlaan 3004, 1081 LA Amsterdam, The Netherlands

**Keywords:** phenothiazines, laser irradiation, photoactivated drugs, photoproducts, hypergravity, LDC, drug stability

## Abstract

Pharmaceuticals carried into space are subjected to different gravitational conditions. Hypergravity is encountered in the first stage, during spacecraft launching. The stability of medicines represents a critical element of space missions, especially long-duration ones. Therefore, stability studies should be envisaged before the implementation of drugs for future deep space travel, where the available pharmaceuticals would be limited and restocking from Earth would be impossible. Multipurpose drugs should be proposed for this reason, such as phenothiazine derivatives that can be transformed by optical methods into antimicrobial agents. Within this preliminary study, promethazine and thioridazine aqueous solutions were exposed to UV laser radiation that modified their structures and generated a mixture of photoproducts efficient against particular bacteria. Subsequently, they were subjected to 20 g in the European Space Agency’s Large Diameter Centrifuge. The aim was to evaluate the impact of hypergravity on the physico-chemical and spectral properties of unirradiated and laser-irradiated medicine solutions through pH assay, UV-Vis/FTIR absorption spectroscopy, and thin-layer chromatography. The results revealed no substantial alterations in centrifuged samples when compared to uncentrifuged ones. Due to their stability after high-g episodes, laser-exposed phenothiazines could be considered for future space missions.

## 1. Introduction

The widespread use of anti-motion sickness (AMS) drugs ensures considerably improved quality of life for individuals, such as motor vehicle passengers, pilots, sailors, and astronauts. Space motion sickness (SMS) is among the foremost challenges encountered during space missions. It can be noticed within the first days after transiting from terrestrial to microgravity conditions [1]. Dating back to such early missions as Russian Vostok 2 and US Apollo 8 programs, crewmembers have experienced impeding medical conditions of SMS [2]. SMS endured by astronauts presents symptoms similar to other types of motion sickness. The triggering environmental conditions seem, though, to be different; SMS sensitivity not being predictable by sensitivity levels associated with influencing factors of terrestrial origins [3]. AMS drugs have been administered over time to crewmembers, the first intramuscular (IM) injection of promethazine (PMZ) with remarkable results taking place during the Space Shuttle STS-29 mission [4]. The efficacy of such treatment exceeded 90% during spaceflight [5,6], and despite concerns regarding PMZ side effects observed on Earth, only a 4.8% incidence of sedation occurred throughout 14 Space Shuttle missions [7]. It has also been indicated that symptoms did not reappear after PMZ administration was interrupted compared to traditional AMS pharmacological treatments [8]. In addition to its IM dispensing, promising results have been found on dogs when treated with an intranasal (IN) PMZ dosage form as a non-invasive alternative. A faster absorption has been obtained via IN administration compared to IM injection, achieving identical bioavailability [9].

Besides the antihistaminic, anticholinergic and antiemetic properties of PMZ [10], other phenothiazine derivatives not only increase the effectiveness of antibiotics but may also possess inherent antibacterial activity [11]. Thioridazine (TZ), for instance, has shown activity against a large number of Gram-positive and Gram-negative bacterial strains [12]. It has been tested in vitro and ex vivo, demonstrating its activity against *Mycobacterium tuberculosis* (Mtb) [13,14]. Antibiotics that have had no success alone in response to extensively drug-resistant Mtb, in combined therapy with TZ contributed to the curing of patients [14,15,16]. Long-term space missions are inevitably accompanied by a multitude of microbial contaminations onboard [17,18], which may lead to serious infections when considering the decreased immune responses of astronauts in the spaceflight environment [19]. Moreover, TZ has also been considered as an antitumoral agent, selectively targeting cancer stem cells but not affecting normal blood stem cells as evidenced in Ref. [20]. When it comes to deep space exploration, the oncogenic potential of galactic cosmic radiation may also compromise astronauts’ health [21].

Space medical kits contain a limited number of pharmaceuticals that may not cover some of the health-threatening situations. More than that, extended space exploratory missions will not be able to conduct pharmaceutical restocking from Earth. Consequently, the implementation of multipurpose drugs may be an appropriate strategy. Thus, already well-characterized and approved drugs could be used, apart from their initial medical indications, to treat other conditions as well. Repurposing non-antibiotic drugs may be a suitable approach for combating antimicrobial resistance [22]. Furthermore, these drugs can be exposed to laser irradiation to produce photoproducts with higher antimicrobial activity than the parent compound. Previous studies emphasized that phenothiazines (considered non-antibiotics) exposed in solution to UV laser radiation acquired improved antimicrobial properties against MDR bacteria due to molecular modifications leading to the generation of photoproducts [23,24,25,26,27,28]. For the above-mentioned reasons, PMZ and TZ may be regarded as valid candidates for space medical kits of future missions because of their multifunctional properties.

However, safe, effective, and stable medicines are an essential part of every mission. It has been evidenced that the stability of pharmaceuticals in spaceflight conditions is compromised [29]. Therefore, before being put into effect, it would be prudent to investigate the impact of various space environmental factors on them (e.g., extreme gravitational and radiation conditions, varying temperature and humidity, excessive vibrations as well as CO_2_-rich atmosphere), even though it is not required by space agencies. The present study focuses on the effect of high-g conditions on unirradiated and laser-irradiated PMZ and TZ. Such tests are sought because the use of pharmaceuticals in microgravity always occurs after they transit through a hypergravity environment (i.e., during spacecraft launching). Moreover, increased gravity can lead to sedimentation in liquid formulations [30], thus possibly augmenting drug instabilities. Even if solid or powdered dosage forms are often regarded to be more stable compared to liquid ones, evidence shows that they may exhibit degradations after long-term stowage in spaceflight conditions [31], thus justifying our study on phenothiazine solutions.

For this hypergravity experiment, the Large Diameter Centrifuge (LDC) of the European Space Agency (ESA) has been used [32]. PMZ and TZ solutions were subjected to 20 times higher gravitational acceleration than at the Earth’s surface to generate some understanding of the effect of high-g levels on our samples. Physico-chemical and spectral properties of the samples were monitored before and after centrifugation, and a comparative investigation was made between them. Our preliminary study provides an insight into the stability of unirradiated and laser-irradiated phenothiazine solutions after hypergravity exposure.

## 2. Materials and Methods

### 2.1. Medicine Solutions

**Figure 1:**
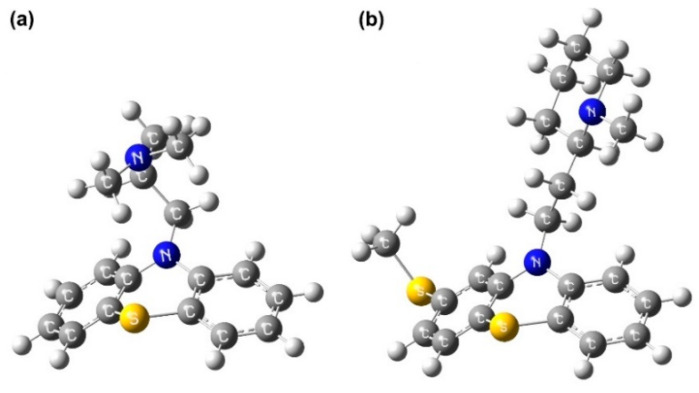
Molecular representation of phenothiazines. Optimized geometry of (**a**) PMZ and (**b**) TZ obtained using Gaussian 09 software (density functional theory with the B3LYP functional and the 6-31G(d,p) basis set) [33].

Phenothiazine derivatives, PMZ (C_17_H_20_N_2_S·HCl) and TZ (C_21_H_26_N_2_S_2_·HCl), were used to produce the medicine solutions in ultrapure water at 2 and 20 mg/mL concentrations. After preparation, solutions were refrigerated (4 °C) to protect them from environmental light and temperature fluctuations. Both compounds were purchased in hydrochloride form as powders with purity ≥ 98% (Sigma-Aldrich, St. Louis, MO, USA). The optimized molecular structures of PMZ and TZ obtained via energy minimization calculations by Gaussian 09 computational chemistry software are displayed in Figure 1.

### 2.2. Laser Irradiation Protocol

A volume of 2 mL from each combination of medicine type (PMZ and TZ), concentration (2 and 20 mg/mL), and irradiation time (1, 15, 30, 60, 120, and 240 min) were exposed successively to an Nd:YAG pulsed laser (266 nm, 6.5 mJ, 10 Hz, 6 ns FWHM), while keeping an unirradiated sample from every solution for comparison. Ref. [34] presents the irradiation protocol and the experimental setup.

The irradiation source and parameters were selected based on previous studies that showed how antipsychotic non-antibiotics can turn into antimicrobial agents within hours [24]. The improved antimicrobial effect of photoactive phenothiazines against MDR bacteria is due to the newly generated photoproducts obtained by exposure to the high-energy UV laser beam, thus obtaining better results with TZ photoproducts than with known antibiotics, for instance [28]. The impact of laser radiation on the stability of PMZ and TZ solutions was studied under terrestrial gravity in Refs. [35,36].

Our study did not intend to simulate space radiation effects on medicine solutions. It aimed instead to provide an insight into the specific properties of UV laser radiation that facilitate the enhancement of the antimicrobial activity of photoresponsive pharmaceuticals to approach MDR-associated challenges.

### 2.3. Hypergravity Exposure Protocol

The LDC, a high-g ground-based simulator, was implemented within this study. It has four rotational arms (each designed with eight standard locking positions) equipped with six experiment-housing gondolas which at a maximum velocity of 67 rpm fully swing-out. In such an operational mode, the LDC reaches a diameter of 8 m, hence the impurity of the gravity produced by the rotating system is minimized. Each gondola of the LDC is equipped with low-frequency industrial ICP accelerometers (626B02, PCB Piezotronics, Depew, NY, USA). A LabVIEW-based user interface ensures a fully programmable facility, where gravity profiles can range from 1 to 20 g. In-depth operating principles and characteristics of the LDC are given in Refs. [32,37].

For this hypergravity experiment, 1 mL from each medicine solution combination was placed in conical black polypropylene centrifuge tubes (Rotilabo) to preserve drug stability, which in turn were positioned in standard microtube racks. To attain the best configuration of the LDC, the racks were installed on the floor inside an outer gondola secured at the extremity of one of the rotor arms. In this way, medicine samples were exposed to the maximum 20 g generated as a result of the terrestrial gravitational acceleration and the centripetal acceleration of the LDC. Subjecting solutions to such a high gravitational level was chosen to speed up the occurrence of possible changes which would have been produced during exposure to lower hypergravity values over a longer period of time. The high-g treatment of samples was performed according to the protocol detailed in Ref. [38], taking into consideration the advantages of the LDC vs. a standard laboratory centrifuge (i.e., diminished inertial shear [39] and Coriolis force [40]). Since such high gravity levels can occur, especially within unmanned missions [41,42], the experiment carried out at 20 g would provide an insight into the behavior of phenothiazine solutions subjected to such an environment.

### 2.4. Medicine Solutions Characterization

**Figure 2:**
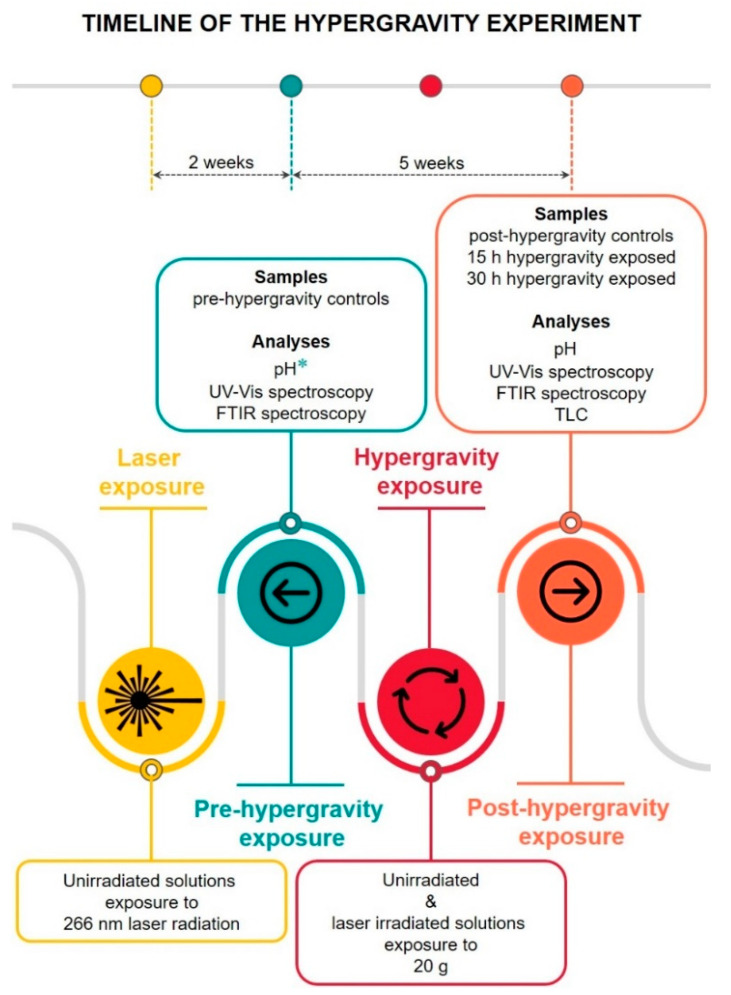
Graphical representation of the hypergravity experiment. The timeline of the study illustrates the various phases together with their associated sets of samples and types of analyses. Each set of samples comprises all combinations of medicine types (PMZ and TZ), concentrations (2 and 20 mg/mL), and irradiation times (1, 15, 30, 60, 120, and 240 min). *  Pre-hypergravity pH data obtained immediately after the laser irradiation process. Note bene: controls—unirradiated and laser-irradiated solutions not subjected to hypergravity conditions.

Our study aimed to examine the impact of hypergravity on unexposed and laser-exposed phenothiazine solutions to provide information regarding their stability after high-g episodes. Samples were investigated by different methods and the measurements were carried out at laboratory temperature (20–25 °C). Although it was not the main purpose of this work, the analyses were first made on control samples pre-hypergravity exposure (i.e., uncentrifuged solutions, both unirradiated and laser-irradiated). The physico-chemical and spectral properties of uncentrifuged solutions were provided so that a subsequent comparison with the centrifuged ones would be possible. Thus, three sets of samples were investigated: (i) uncentrifuged, (ii) 15 h, and (iii) 30 h centrifuged solutions. Each set included all combinations of medicine types, concentrations, and irradiation times. Figure 2 presents the sets of samples and types of analyses while following the different phases and timelines of the experiment. To explore drug stability, samples were kept for several weeks after exposure to 20 g. The interval between pre- and post-hypergravity measurements was 5 weeks.

#### 2.4.1. pH Assay

The hydrogen ion concentration of phenothiazine samples was measured in triplicate using a pH meter (Lab 860, Schott Instruments, Mainz, Germany) with an accuracy of ±0.015.

#### 2.4.2. UV-Vis Absorption Spectroscopy

The absorption spectra of PMZ and TZ solutions were recorded using a 1 mm thick spectrophotometric cell between 200 and 1200 nm with a UV-Vis-NIR spectrophotometer (Lambda 950, PerkinElmer, Waltham, MA, USA) at a resolution of 1 nm. The ±2.174% overall measuring error has been taken into account, as described in Ref. [34].

#### 2.4.3. FTIR Absorption Spectroscopy

The experimental IR spectra of drug solutions were registered from 4000 to 400 cm^−1^ at 4 cm^−1^ resolution by an FTIR spectrometer (Nicolet iS50, Thermo Scientific, Waltham, MA, USA). To avoid high absorption of the aqueous solutions, samples were dried on a KRS-5 thallium bromo-iodide optical crystal, applying 20 μL drops at a time. The background of the crystal was subtracted from the final spectra.

The theoretical IR spectra of TZ and PMZ were calculated using Gaussian 09 software [33]. First, the molecular structures were subjected to geometry optimization followed by the calculation of vibrational wavenumbers using the density functional theory. The hybrid functional B3LYP method with a 6-311G(d,p) basis set was used. To obtain the scaled vibrational wavenumbers, the 7 × 10^−6^ × υ_calc_^2^ + 0.9783 × υ_calc_ + 12.68 quadratic scaling function was implemented [43].

#### 2.4.4. Thin-Layer Chromatography

Thin-layer chromatography (TLC) allowed separation and comparison of photoreaction products formed after the 4 h irradiation process of PMZ and TZ solutions. The stationary phase consisted of aluminum oxide silica gel-coated plates (60 F_254,_ Merck, Darmstadt, Germany), while the mobile phase had a 50:50:1 volume ratio of a mixture of acetone:methanol:25% ammonia solution. From the unirradiated and laser-irradiated phenothiazine samples, 1 μL drops were added onto the TLC plate, and the solvent (i.e., ultrapure water) was allowed to evaporate. The plate was then placed into the developing tank, where a filter paper maintained a saturated vapor atmosphere. Once developed, plates were examined under 365 nm/254 nm by a UV illumination cabinet (C-65 Chromato-Vue, UVP, Upland, CA, USA) and photographed with a digital single-lens reflex camera (D80, Nikon, Tokyo, Japan). For evaluation, the JustTLC (Sweday, Södra Sandby, Sweden) image analysis software was utilized.

## 3. Results and Discussion

### 3.1. pH Analysis

To establish the impact of hypergravity on the acidity of unirradiated and UV-irradiated samples, the hydrogen ion concentration of PMZ and TZ was monitored pre- and post-centrifugation. During the 240 min UV irradiation process, the pH of 2 and 20 mg/mL solutions exhibited exponential decay as the laser irradiation time increased. The most considerable pH drop was noticed at the lower concentration for both phenothiazines, pH of control samples before the hypergravity experiment ranging from 4.99 to 2.86 in the case of PMZ and from 5.07 to 2.90 for TZ. At the higher concentration, the pH of PMZ diminished from 4.76 to 3.04, while for unirradiated TZ the pH was 4.45 decreasing to 3.29 after 4 h of exposure (Supplementary Table S1).

A comparison was made between the pH values of uncentrifuged and centrifuged solutions. Centrifuged PMZ showed slightly increased pH levels at 2 mg/mL concentration compared to pre-hypergravity controls, especially when the samples were exposed to UV for longer time intervals (Supplementary Figure S1a), while at 20 mg/mL it exhibited similar values before and after the increased gravity exposure (Supplementary Figure S1b). TZ samples at 2 mg/mL exposed to increased gravitational acceleration proved to have slightly more elevated pH levels (particularly the 30 h centrifuged solutions) than pre-hypergravity measured controls. However, it was also observed that starting from 1 h of irradiation time upwards, values of 15 h centrifuged and uncentrifuged samples obtained post-hypergravity overlap (Supplementary Figure S1c). As for 20 mg/mL TZ, lower pH values were noticed after the LDC campaign than before it, especially for shorter laser interaction times (Supplementary Figure S1d).

Consequently, one can state that the rise in pH levels measured post-hypergravity in contrast to values registered pre-hypergravity experiment may be due to the aging effect experienced by medicine solutions over time which, most probably, induces the acidity loss (except the pH decrease in the case of 20 mg/mL TZ). Although, samples subjected to high-g treatment remained stable within measurement error limits with respect to values obtained for uncentrifuged solutions measured after the LDC experiment. The pH difference is already discernible between pre- and post-hypergravity controls. This may be owing to the presence of transient photoproducts generated throughout the irradiation process (existing in solutions only 24–48 h after laser exposure [36,44]), absent at the time of measurements performed after the hypergravity campaign.

The exponential pH decay experienced throughout the 4 h irradiation process is caused by the hydrogen ion concentration increase, which may be explained by the photoionization taking place during laser exposure. It has to be mentioned that the pH of solutions may influence the photodegradation rate since pH affects quantum yields and there is a dependence of degradation rates on quantum yields, as discussed in Ref. [45]. The photodegradation process of phenothiazines has been studied in Refs. [46,47], a phototransformation pathway being proposed for TZ (simulated sunlight irradiation via UV-Vis xenon lamp, initial conditions: 800 mL ultrapure water solutions, 0.5 and 50 mg/L concentrations, 6.5 pH, 20 ± 2 °C temperature) in Ref. [48]. Wilde et al. reported that the primary elimination of TZ follows the two-steps first-order exponential kinetic model. The molecular structure of the identified photoproducts was clarified by liquid chromatography-mass spectrometry (LC-MS), where the degradation pathway included hydroxylation, sulfoxidation, dehydroxylation, as well as S- and N-dealkylation, TZ-2-sulfoxide and TZ-5-sulfoxide being the two main occurring products known as the pharmacologically active human metabolites of TZ [48]. Out of the nine photoproducts, visualized by previous TLC analysis [28], four were identified following the irradiation of TZ under the same conditions as in the present study (4 h 266 nm laser exposure, 2 mg/mL concentration, 20–25 °C temperature), namely: mesoridazine (TZ-2-sulfoxide) which suffers further 2-oxidation to sulforidazine (TZ-2-sulphone), TZ-5-sulfoxide and TZ-N-desmethyl [28].

### 3.2. UV-Vis Absorption Analysis

**Figure 3:**
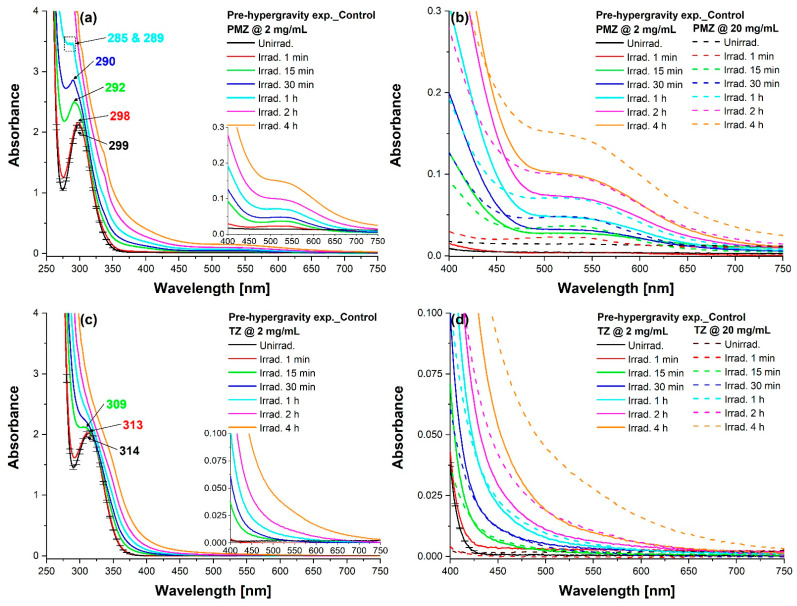
UV-Vis absorption spectra of uncentrifuged phenothiazine solutions recorded prior to the hypergravity treatment. (**a**) Unexposed and laser-exposed 2 mg/mL PMZ. (**b**) Comparison of 2 and 20 mg/mL PMZ. (**c**) Unirradiated and UV-irradiated 2 mg/mL TZ. (**d**) Comparison of 2 and 20 mg/mL TZ. Error bars: ±2.174%.

On one hand, this study revealed the impact of the 266 nm laser irradiation process on the UV-Vis absorption spectra of PMZ and TZ solutions, when measurements were carried out before hypergravity exposure. Modifications can be observed in Figure 3 as the interaction time with laser radiation increases.

At 2 mg/mL, unirradiated PMZ has an absorption band peaking at 299 nm and unirradiated TZ at 314 nm, which can be attributed to n–π* electronic transition [35]. By extending the UV exposure time interval, the respective peaks suffered hypso- and hyperchromic shifts, an exponential decay characterizing the kinetics of the peak wavelength and a linear increase the kinetics of the absorbance intensity in the case of PMZ as well as TZ (Supplementary Figure S2). PMZ experienced a split of the absorption maximum into two peaks (285 and 289 nm) corresponding to the 1 h irradiated sample and a 9 nm hypsochromic shift during the first 30 min of laser exposure (Figure 3a). Within 475–625 nm, a new broader band emerged, featuring a slight hyperchromic effect throughout the 4 h irradiation (Figure 3a inset). In the same Vis spectral region, a similar behavior (new broadband formation and gradual intensity increase up to 4 h exposure) was noticed in the case of 20 mg/mL PMZ (Figure 3b). When laser exposure of 2 mg/mL TZ took place, a 5 nm hypsochromic shift of the absorption peak was detected by the end of the 15 min irradiation alongside a hyperchromic effect (Figure 3c). As for the 20 mg/mL concentration, progressive hyperchromic shifts were observed as laser irradiation time increased (Figure 3d).

On the other hand, the focus was on the effect of different gravitational conditions on the evolution of UV-Vis absorption spectra of the above-mentioned drug solutions, measurements being performed before and after the exposure to hypergravity. An absorbance intensity decline was noticed as the prevalent trend among 2 mg/mL samples subjected to hypergravity conditions (Supplementary Figures S3 and S4). After centrifugation, unirradiated PMZ and TZ experienced a 1 nm hypsochromic shift of their characteristic peak in addition to the maximum 4.8 and 5.1% hypochromic shift, respectively, when pre-hypergravity controls and hypergravity treated samples were compared (Supplementary Figures S3a and S4a). Regarding irradiated solutions, the lowest absorbance was associated mainly with the shorter centrifugation time in the case of PMZ and with the longer one for TZ. As laser interaction time increased, spectra of the high-g exposed samples fitted outside the error bars limit (Supplementary Figure S3d–g); however, even post-hypergravity controls showed decreased intensity at prolonged laser exposure (Supplementary Figure S3f–g). Spectra of hypergravity treated 20 mg/mL PMZ solutions presented hyperchromic shifts, the more the irradiation time increased, the smaller discrepancy was observed between uncentrifuged and centrifuged samples (Supplementary Figure S5). The 4 h irradiated PMZ remained within the error limits regardless of the gravitational conditions and measurements timeframe (Supplementary Figure S5g). Considering 20 mg/mL TZ, UV-Vis spectra of unirradiated and laser-irradiated control solutions registered post-centrifugation exhibited hyperchromic effects, whereas high-g exposed samples suffered mostly hypochromic shifts, the 30 h centrifuged ones having the lowest intensity in all the cases (Supplementary Figure S6).

The choice for the laser exposure of 2 mg/mL phenothiazine solutions can be attributed to the fact that at, a 6.5 mJ energy level, the parent compound rapidly transforms into new photoproducts [24]. Moreover, at 2 mg/mL the contribution of micelles in solutions can be avoided, being below the critical concentration [49]. In contrast, at 20 mg/mL, micelle formation may take place in the first steps of the irradiation process as suggested in the case of chlorpromazine by Pascu et al. Moreover, at such a high concentration, the number and amounts of generated photoproducts are enhanced [24].

The band in the UV region in the case of 2 mg/mL concentration may appear as a result of the sulfur lone-electron pairs in the phenothiazine ring [50]. Its hypsochromic shift in such a polar solvent as water, alongside its low molar attenuation coefficient (relative to the high values of the peak at 249 and 262 nm observed for 0.2 mg/mL diluted PMZ [35] and TZ samples [28], respectively) substantiate the n–π* nature of the band, noting that it can also be contingent on the substituents (substitution at the phenothiazine 2-position rather than nitrogen substitution by the 10-dimethylaminopropyl group) [50]. The hypso- and hyperchromic shifts encountered throughout the 4 h irradiation process, evidenced in Figure 3, imply that modifications in the UV-Vis spectra are due to the photodegradation of parent compounds (PMZ and TZ). This degradation alters the initial chemical structure by including functional groups that, in turn, lead to the generation of new reaction products.

Regarding the hypergravity exposure of unirradiated and laser-irradiated solutions, changes in the absorbance intensity have been observed for centrifuged samples without the appearance of new bands. In addition, a slight 1 nm blue shift was also noticed in 2 mg/mL unirradiated high-g treated samples compared to controls before and after the hypergravity experiment. However, it is noteworthy to point out that even post-hypergravity control solutions suffered hypo- or hyperchromic shifts in contrast to pre-hypergravity controls. An explanation may reside in the fact that, within the time interval between pre- and post-hypergravity measurements, aging effects may occur in control drug solutions too, without being subjected to increased gravitational conditions.

### 3.3. FTIR Analysis

**Figure 4:**
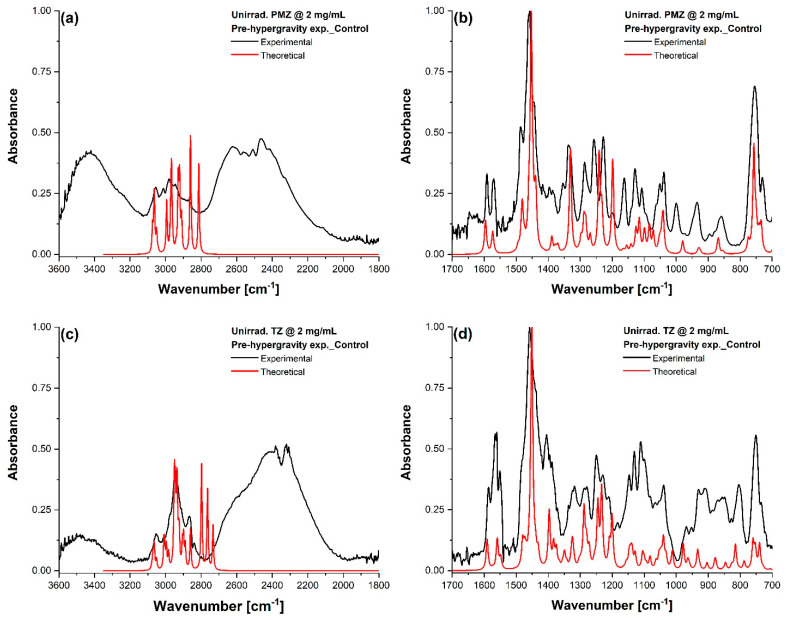
Experimental and theoretical FTIR spectra of unirradiated phenothiazine solutions. Unirradiated PMZ at 2 mg/mL in the range (**a**) 3600–1800 cm^−1^ and (**b**) 1700–700 cm^−1^. Unirradiated TZ at 2 mg/mL in the range (**c**) 3600–1800 cm^−1^ and (**d**) 1700–700 cm^−1^.

FTIR spectroscopy was used to investigate the changes in the molecular structure of UV-irradiated phenothiazines before and after hypergravity exposure. First, the theoretical IR characteristic vibrations of unirradiated PMZ and TZ were calculated and were utilized to interpret the experimental results of the FTIR absorption spectra. For PMZ (Figure 4a,b) and TZ (Figure 4c,d), the IR spectra of assigned normal vibrations were plotted against the scaled theoretical ones for comparison. The characteristic vibrational frequencies of both phenothiazines were identified using Gaussian 09 software (Supplementary Tables S2 and S3). Furthermore, the motion associated with the vibration (vectors on each atom) for every wavenumber is depicted in the Supplementary Materials (Supplementary Figures S7 and S8).

**Figure 5:**
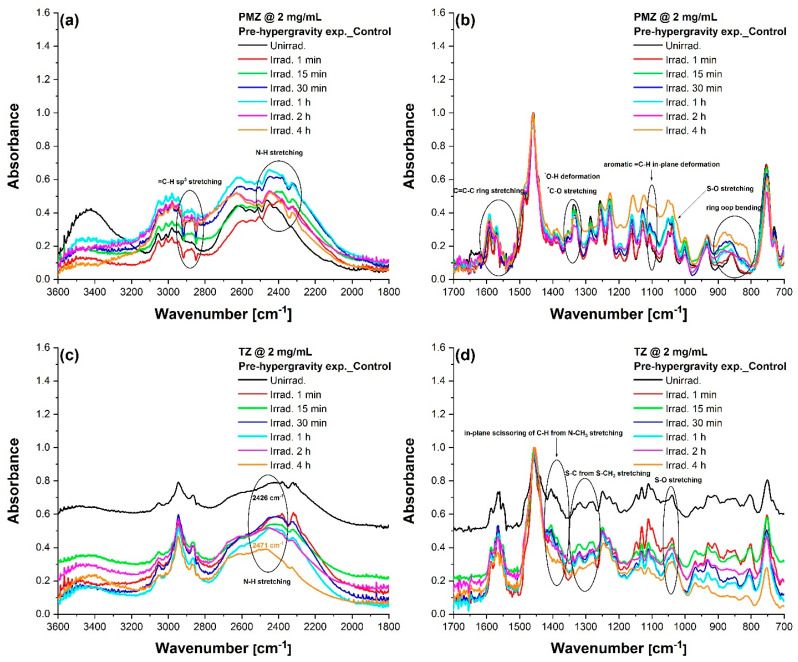
FTIR absorption spectra of unirradiated and UV-irradiated phenothiazine solutions. PMZ samples at 2 mg/mL registered before the hypergravity experiment in the range (**a**) 3600–1800 cm^−1^ and (**b**) 1700–700 cm^−1^. TZ samples at 2 mg/mL in terrestrial gravitational conditions recorded in the frequency domain (**c**) 3600–1800 cm^−1^ and (**d**) 1700–700 cm^−1^ before the high-g treatment.

Subsequently, the IR spectra of irradiated solutions were compared to the unirradiated ones to identify modifications to the parental molecular structures (PMZ and TZ). Figure 5a,b shows the IR spectra of unirradiated and irradiated PMZ, while Figure 5c,d shows the IR spectra of TZ, the samples being examined pre-hypergravity exposure.

The formation of new compounds during the 266 nm laser exposure of 2 mg/mL PMZ was emphasized by the disappearance of the 1336 cm^−1^ band and the emergence of new ones at 1338 cm^−1^, corresponding to the 120 min irradiated sample (Figure 5a,b). This was attributed to the combination of O-H deformation vibration and C-O stretching vibration of phenol, thus revealing the formation of a hydroxyl photoproduct. Generation of the respective hydroxyl photoproduct was confirmed by the presence of a new band at 818 cm^−1^ associated with the out-of-plane deformation vibration of two neighboring aromatic H atoms and by the new band at 1579 cm^−1^ representing the C=O stretching vibration of a phenol group. The new band at 1025 cm^−1^ was caused by the stretching vibration of the S-O bond, thus pointing out the generation of oxidized compounds.

The appearance of the band at 1405 cm^−1^ in the unirradiated TZ spectrum was attributed to the deformation vibration of C-H (N/\−CH3) which did not occur after 4 h laser exposure, suggesting the cleavage of the N-CH_3_ bond (Figure 5c,d). Following 2 h UV irradiation, a decrease in intensity was noticed by changing the shape of the 1317 cm^−1^ band, corresponding to symmetric stretching vibration of the S-C bond from S-CH_3_, thus also implying cleavage. The emergence of a new band at 1050 cm^−1^, attributed to the S-O bond stretching vibration of sulfoxide, indicated the generation of an oxidative photoproduct. Additionally, a 4 nm bathochromic shift suggested the generation of new photoproducts. The disappearance of the 1404 cm^−1^ band from the unexposed TZ spectrum and the occurrence of a new band at 1385 cm^−1^ in the 4 h irradiated spectrum (characterized by the O-H bond deformation vibration and the stretching vibration of the C-O bond within a phenol group) implied the attachment of such groups to the molecular structure of TZ.

As both PMZ and TZ have chiral centers, some of the photoproducts generated during irradiation can be isomeric forms of the parental compound [51,52], a process known as photoisomerization [45]. Future studies are recommended to use LC-MS to identify the molecular structures based on high-resolution mass spectra and fragmentation patterns. Chromatographic peaks with different retention times described by similar fragmentation patterns and identical nominal m/z are expected [48].

In conclusion, FTIR spectra of phenothiazines indicated modifications at the molecular level of parental compounds (PMZ and TZ), pointing out that photoproducts containing phenol and sulfoxide groups have been generated. These modifications could be observed exclusively for 2 mg/mL solutions exposed to UV laser radiation. In the case of 20 mg/mL samples, only the characteristic vibrations of molecular bonds can be noticed due to the high concentration of PMZ and TZ in the solution after exposure to laser radiation compared to the rest of the photoproducts, thus influencing the FTIR spectra.

Regarding the evolution of uncentrifuged and centrifuged phenothiazines, one may observe that at the lower concentration both medicines exhibited modifications when comparing pre-hypergravity with post-hypergravity spectra (Supplementary Figures S9 and S10). Changes appeared even between control solutions registered before and after the LDC campaign, which are most likely due to a slight degradation of the samples in the considered time interval. Concerning the hypergravity treated higher concentration solutions, neither unexposed nor laser-exposed medicines showed FTIR spectra modifications after being subjected to 20 g conditions. This implies that both phenothiazines continued to remain stable despite the 15 and 30 h centrifugation (Supplementary Figures S11 and S12).

### 3.4. TLC Analysis

The evaluation of unirradiated and irradiated phenothiazine solutions subjected to 20 g was possible also through TLC image analysis. This implies the qualitative and quantitative analysis of the parental compounds (PMZ and TZ), as well as their photoproducts resulting from the 4 h irradiation process. The qualitative analysis involved the visualization, identification, and direct comparison of the samples, whereas the quantitative evaluation provided their relative volumes. Solutions at 2 mg/mL appeared to comprise photoproducts at not high enough concentration, consequently only the 20 mg/mL samples have been quantified by TLC.

**Figure 6:**
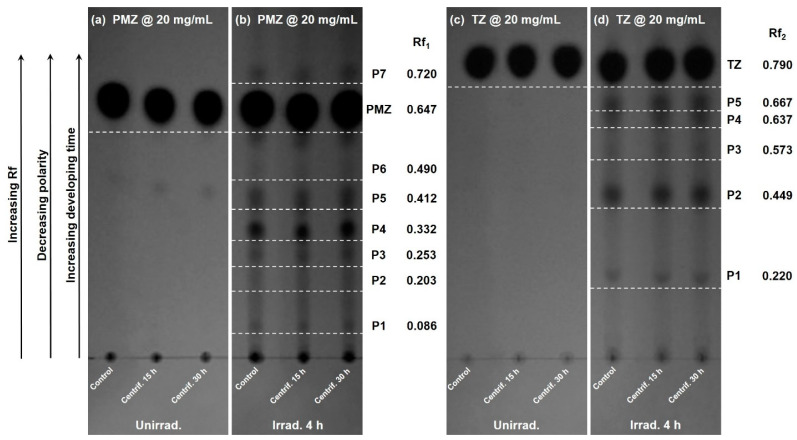
TLC plates with 20 mg/mL phenothiazines. Migration and separation of (**a**) unexposed PMZ, (**b**) 4 h laser-exposed PMZ, (**c**) unirradiated TZ, and (**d**) 4 h irradiated TZ after the hypergravity experiment, each featuring three columns of uncentrifuged, 15 and 30 h centrifuged samples. Rf_1_—retention factors for CPZ and its photoproducts, Rf_2_—retention factors for PZ and its photoproducts. Rf of photoproducts increases with the developing time, while their polarity decreases (arrow indications).

Figure 6 represents TLC plates visualized at 254 nm, photographed, and presented as greyscale images. The analysis showed unirradiated PMZ, separation of the photoproducts for 4 h irradiated PMZ, unirradiated TZ, and separation of the photoproducts for 4 h irradiated TZ, all of them being subjected to terrestrial gravitation, hypergravity for 15 and 30 h, respectively. Thus, the separation and migration of the photoproducts function of their polarity are displayed in sets of three columns.

TLC image analysis is faster, more cost-effective, and it can evaluate simultaneously all the samples compared to the slit scanning densitometry technique [53,54,55]. The images in Figure 6 were uploaded into the JustTLC image analysis software which can calculate the area of spots by comparing their intensity to that of the surrounding background. Within each band region, the volume is computed by summing the variations between the estimated background and the noise-filtered input image [56], relying on the presumption that the intensity and the spot area are as a function of concentration [54].

The retention factor (Rf), defined as the ratio of the distance the spot (i.e., compound) moved through the stationary phase above the origin (starting line, where the medicine droplets were applied) to the distance the solvent front traveled above the origin, was determined by considering the origin to be zero and the solvent frontline one. The Rf values obtained for the parental compounds and their photoproducts are indicated in Figure 6. In the case of PMZ, Figure 6a,b emphasizes the presence of seven photoproducts after the 4 h laser exposure, six of them being more polar and one less polar than PMZ, their polarity experiencing a rise from top to bottom. For TZ, five photoproducts were identified, all of them being more polar than TZ, as depicted in Figure 6c,d.

**Figure 7:**
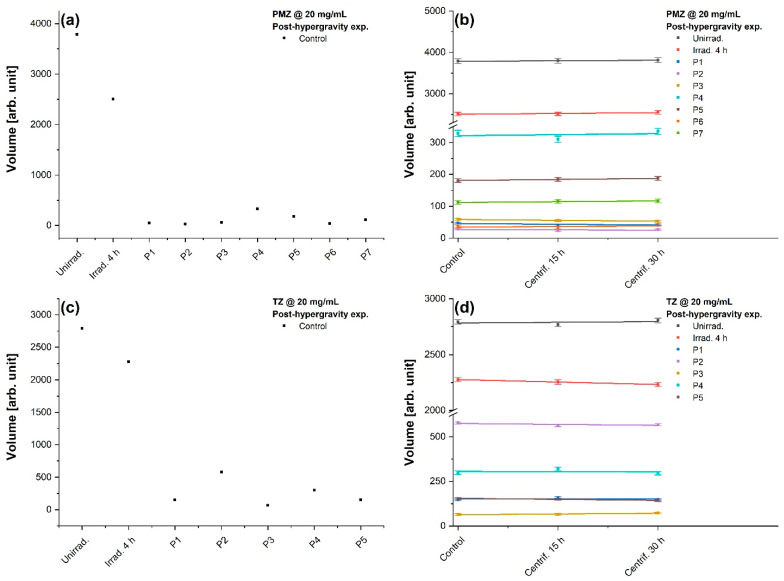
Analysis of TLC plates by JustTLC performed after the hypergravity experiment. (**a**) The volume of PMZ, remaining PMZ after the 4 h laser exposure process, and its seven photoproducts. (**b**) Evolution of uncentrifuged, 15 and 30 h centrifuged PMZ, and its photoproducts volumes. (**c**) The volume of TZ, remaining TZ following 4 h of laser irradiation, and its five generated photoproducts. (**d**) Evolution of TZ and its photoproducts volumes under terrestrial and hypergravity conditions. Error bars: standard deviation.

Figure 7a,b displays the extracted volume via JustTLC analysis for unirradiated PMZ, remaining PMZ in the 4 h irradiated solution, and the photoproducts generated during the irradiation process. The volume of PMZ dropped by 33.7% after 4 h laser exposure as shown in Figure 7a. As for its photoproducts, P4 has the highest volume followed by P5, P7, P3, P1, and P6, the lowest being exhibited by P2. It can be observed in Figure 7b that PMZ and its photoproducts remained stable in time, their volumes staying unchanged regardless of subjection to hypergravity conditions. Concerning TZ, Figure 7c illustrates similarly the volume of TZ and its photoproducts, the initial value of TZ declining by 18.5% after the 4 h irradiation process. With respect to its photoproducts, P2 has the highest volume followed by P4, P5, and P1, P3 having the lowest one. As in the case of PMZ, TZ and its photoproducts indicated proper time stability, meaning that no modifications were observed in their volumes after centrifugation (Figure 7d). The volume levels, in both cases, were within the error limits as depicted in Figure 7b,d.

Overall, it was noticed for both, 20 mg/mL PMZ and TZ, that they are not completely photodegraded after the end of the laser exposure process. Moreover, no differences regarding the volume or number of photoproducts were observed for unirradiated and laser-irradiated solutions subjected to different gravitational conditions. Even between uncentrifuged and centrifuged samples, no modifications were noticed after TLC evaluation.

## 4. Conclusions

The progress of exploratory missions beyond low Earth orbit and even into deep space may cause increased susceptibility to infections and may lead to more severe diseases. Therefore, various antimicrobials (i.e., antibacterial, -fungal, -viral, -parasitic drugs) are currently included in space medical kits, thus allowing a broad range of infections to be counteracted, treated, and contained via different routes of administration. Despite those actions, astronauts’ health may be compromised due to changes in human and microbe physiology as well as by alterations in physico-chemical properties of pharmaceuticals in the spaceflight environment.

The unique environmental factors encountered in space, including different gravitational conditions, may affect drug stability. For this reason, our preliminary investigation focused on the stability of physico-chemical and spectral properties of medicines in increased gravity. The study showed the stability of unirradiated and laser-irradiated PMZ and TZ aqueous solutions after being subjected to a gravitational acceleration 20-fold that experienced on Earth. No significant changes between control and hypergravity-treated samples were evidenced. This is a positive finding, considering their possible implementation in future space missions. One of the first steps towards such a goal is to know if these medicines would remain stable after transiting hypergravity environments, during launch or landing on another planet, for instance.

Since the available pharmaceuticals may be restricted and restocking from Earth may be limited or even inaccessible in some missions, multifunctional medicines would be needed. One strategy may be the repurposing of well-characterized and approved medicines. They may receive by exposure to laser radiation new properties that would allow treatment of different health problems (other than those for which they were initially conceived). Following the irradiation, the photoproducts must be isolated, identified, registered, and approved by the FDA before they can be given to astronauts. Our study did not aim to mimic the impact of space radiation on phenothiazine solutions but invoked instead the specific properties of UV laser radiation to boost the antimicrobial effect of non-antibiotics as a means to tackle MDR-related challenges.

## Data Availability

Data available on request.

## Supplementary Materials

The following supporting information is available online, Table S1: Evolution of the pH of phenothiazine solutions during laser exposure; Figure S1: Evolution of pH of phenothiazine solutions as a function of laser exposure time; Figure S2: Absorption spectra behavior during the UV laser exposure process; Figure S3: Evolution of UV-Vis absorption spectra of 2 mg/mL PMZ solutions exposed to 1 and 20 g gravitational conditions; Figure S4: Evolution of UV-Vis absorption spectra of 2 mg/mL TZ solutions subjected to terrestrial and hypergravity conditions; Figure S5: Evolution of UV-Vis absorption spectra of 20 mg/mL PMZ solutions exposed to 1 and 20 g gravitational conditions; Figure S6: Evolution of UV-Vis absorption spectra of 20 mg/mL TZ solutions subjected to terrestrial and hypergravity conditions; Table S2: Characteristic vibrational frequencies of the molecular bonds of PMZ; Table S3: Characteristic vibrational frequencies of the molecular bonds of TZ; Figure S7: Displacement vectors (blue arrows) illustrating the motion associated with the IR vibrations for the representative wavenumbers from unirradiated PMZ IR spectra; Figure S8: Displacement vectors (blue arrows) showing the motion associated with the IR vibrations for the representative wavenumbers from unirradiated TZ IR spectra; Figure S9: Evolution of FTIR absorption spectra of 2 mg/mL PMZ solutions pre- and post-hypergravity; Figure S10: Evolution of FTIR absorption spectra of 2 mg/mL TZ solutions subjected to 1 and 20 g conditions; Figure S11: Evolution of FTIR absorption spectra of 20 mg/mL PMZ solutions exposed to terrestrial and hypergravity conditions; Figure S12: Evolution of FTIR absorption spectra of 20 mg/mL TZ solutions subjected to 1 and 20 g conditions.

## Sample Availability

Unirradiated and laser-irradiated PMZ and TZ samples are available from the authors.
