# Supplementary material for: Stability Studies of UV Laser Irradiated Promethazine and Thioridazine after Exposure to Hypergravity Conditions
Source: Molecules. 2022 Mar 7;27(5):1728. doi: 10.3390/molecules27051728 (PMC8911845; doi:10.3390/molecules27051728)
Supplement: Supplementary file 1 [file molecules-27-01728-s001.zip › molecules-1569594-supplementary.pdf]

Evolution of the pH of phenothiazine solutions during laser exposure

Table S1. Uncentrifuged and centrifuged solutions at 2 and 2 mg/mL, measured pre- and post-hypergravity treatment.

Drug solution	Concentr. [mg/mL]	Irrad. time [min]	pH			
			Pre-hypegravity Control	Post-hypegravity Control	Post-hypegravity Centrif. 15 h	Post-hypegravity Centrif. 30 h
PMZ	2	0	4.99	4.72	4.81	4.65
		1	4.10	4.06	4.07	4.05
		15	3.61	3.60	3.62	3.63
		30	3.39	3.44	3.42	3.45
		60	3.18	3.25	3.27	3.29
		120	2.98	3.04	3.10	3.08
		240	2.86	2.95	2.95	2.96
	20	0	4.76	4.53	4.51	4.50
		1	4.45	4.33	4.25	4.29
		15	3.73	3.66	3.65	3.73
		30	3.53	3.50	3.53	3.52
		60	3.32	3.34	3.38	3.38
		120	3.20	3.21	3.24	3.24
		240	3.04	3.08	3.13	3.10
TZ	2	0	5.07	4.55	4.56	4.55
		1	4.55	4.48	4.39	4.44
		15	3.84	3.83	3.78	3.90
		30	3.68	3.67	3.63	3.75
		60	3.47	3.47	3.48	3.53
		120	3.19	3.26	3.26	3.29
		240	2.90	2.97	3.01	3.05
	20	0	4.45	4.31	4.42	4.36
		1	4.41	4.25	4.32	4.32
		15	4.09	3.96	3.95	4.00
		30	3.97	3.84	3.85	3.90
		60	3.73	3.60	3.68	3.64
		120	3.61	3.51	3.56	3.55
		240	3.29	3.22	3.26	3.29

Evolution of the pH of phenothiazine solutions as a function of laser exposure time

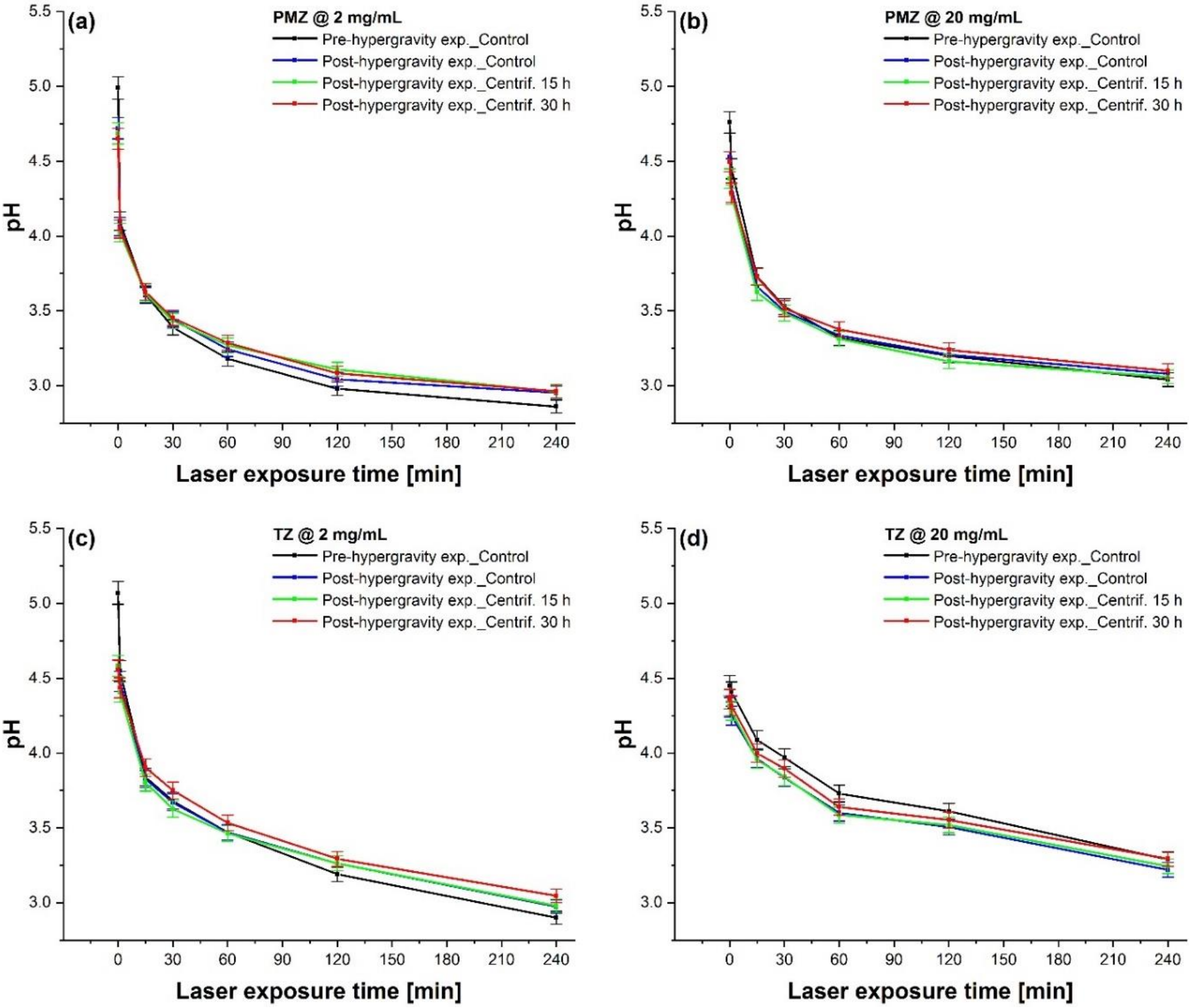

Figure S1. Uncentrifuged and centrifuged (**a**) 2 mg/mL PMZ, (**b**) 20 mg/mL PMZ, (**c**) 2 mg/mL TZ and (**d**) 20 mg/mL TZ solutions measured before and after the hypergravity experiment. Error bars: ± 1.5 %.

Following the 4 h laser exposure, a 42.7 and 36.1% decrease was noticed before centrifugation in 2 and 20 mg/mL PMZ samples, respectively (black curve in Figure S1a and S1b). Analyzing the same uncentrifuged PMZ solutions after the high-g experiment, a 37.5% reduction in the case of the lower concentration and 32% for the higher concentration was determined (blue curve in Figure S1a and S1b), resulting in a 5.2 and 4.1% pH difference between pre- and post-hypergravity measurements. The pH decline is significant already after the first minute of irradiation, particularly for 2 mg/mL samples, values dropping by 17.8% when evaluated prior to the centrifugation experiment and by 14% when registered after it. Even though the pH discrepancy was more pronounced for 2 mg/mL PMZ solutions (i.e., 3.8%), a 2.1% difference was calculated in case of 20 mg/mL concentration.

Control TZ solutions at their lower concentration indicated pre-centrifugation an equivalent pH decrease (i.e., 42.8%) to 2 mg/mL PMZ throughout the 4 h laser interaction, whereas TZ at the higher concentration diminished only by 26.1% (black curve in Figure S1c and S1d). Once measured post-centrifugation, control TZ samples presented a 34.7 and 25.3% pH drop for 2 and 20 mg/mL, respectively (blue curve in Figure S1c and S1d), meaning an 8.1 and 0.8% difference when comparing pre- and post-hypergravity controls.

Absorption spectra behavior during the UV laser exposure process

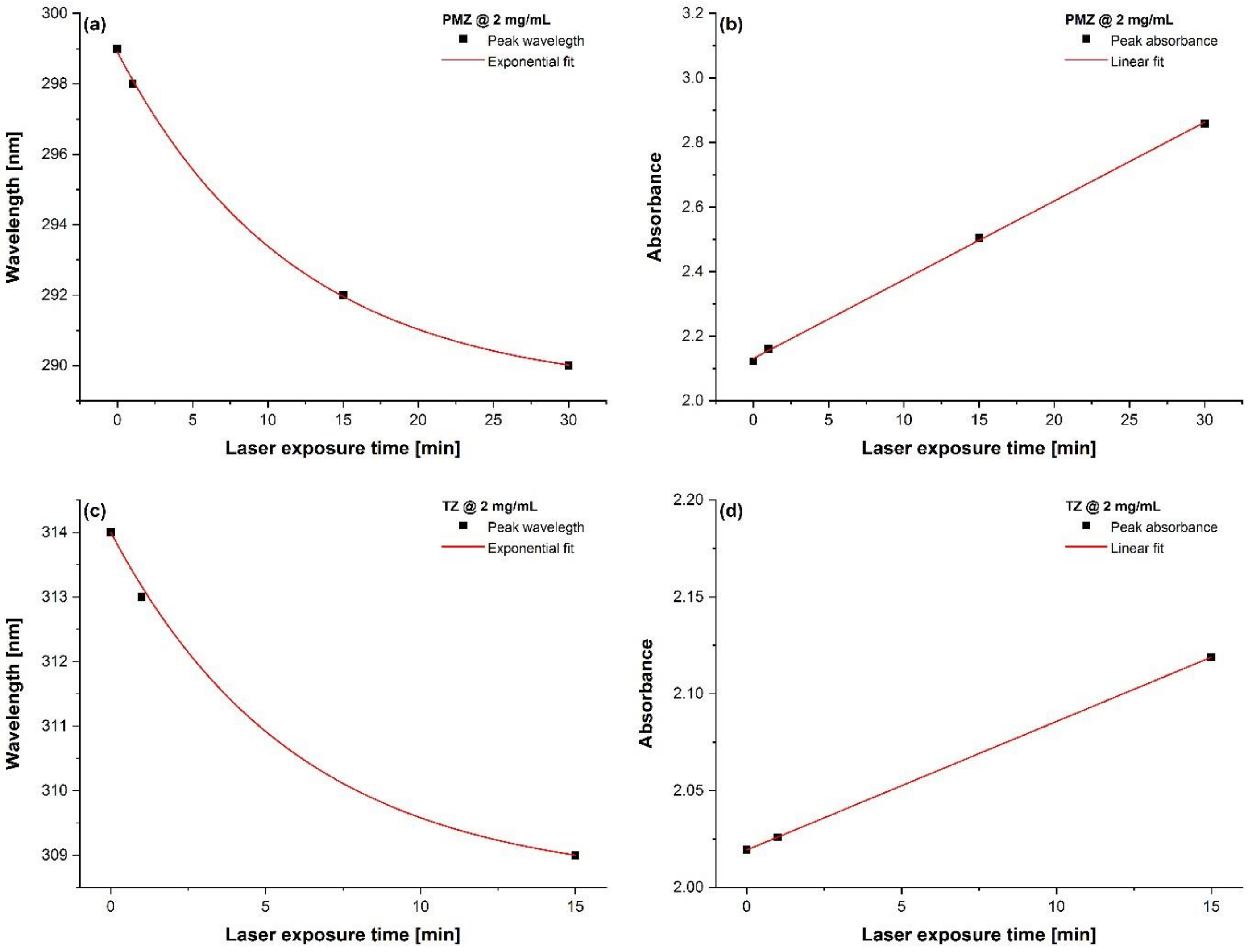

Figure S2. Kinetics of (**a**) peak wavelength and (**b**) peak absorbance of 2 mg/mL PMZ up to 30 min irradiation. Kinetics of (**c**) peak wavelength and (**d**) peak absorbance of 2 mg/mL TZ up to 15 min irradiation.

With the extension of the UV exposure time, the absorption peak of 2 mg/mL PMZ underwent hypso- and hyperchromic shifts, an exponential decay characterizing the kinetics of the peak wavelength (Figure S2a) along with an absorbance intensity raise of 25.8% after 30 min irradiation (Figure S2b). When laser exposure of 2 mg/mL TZ occurred, a 5 nm hypsochromic shift of the absorption maximum was noticed by the end of the 15 min irradiation alongside a hyperchromic shift. The peak wavelength has an exponential decreasing tendency (Figure S2c), similar to PMZ. The kinetics of the absorption maximum follow a linear increase, as the laser exposure time reaches 15 min the upsurge being of 4.7% (Figure S2d).

Evolution of UV-Vis absorption spectra of 2 mg/mL PMZ solutions exposed to 1 and 20 g gravitational conditions

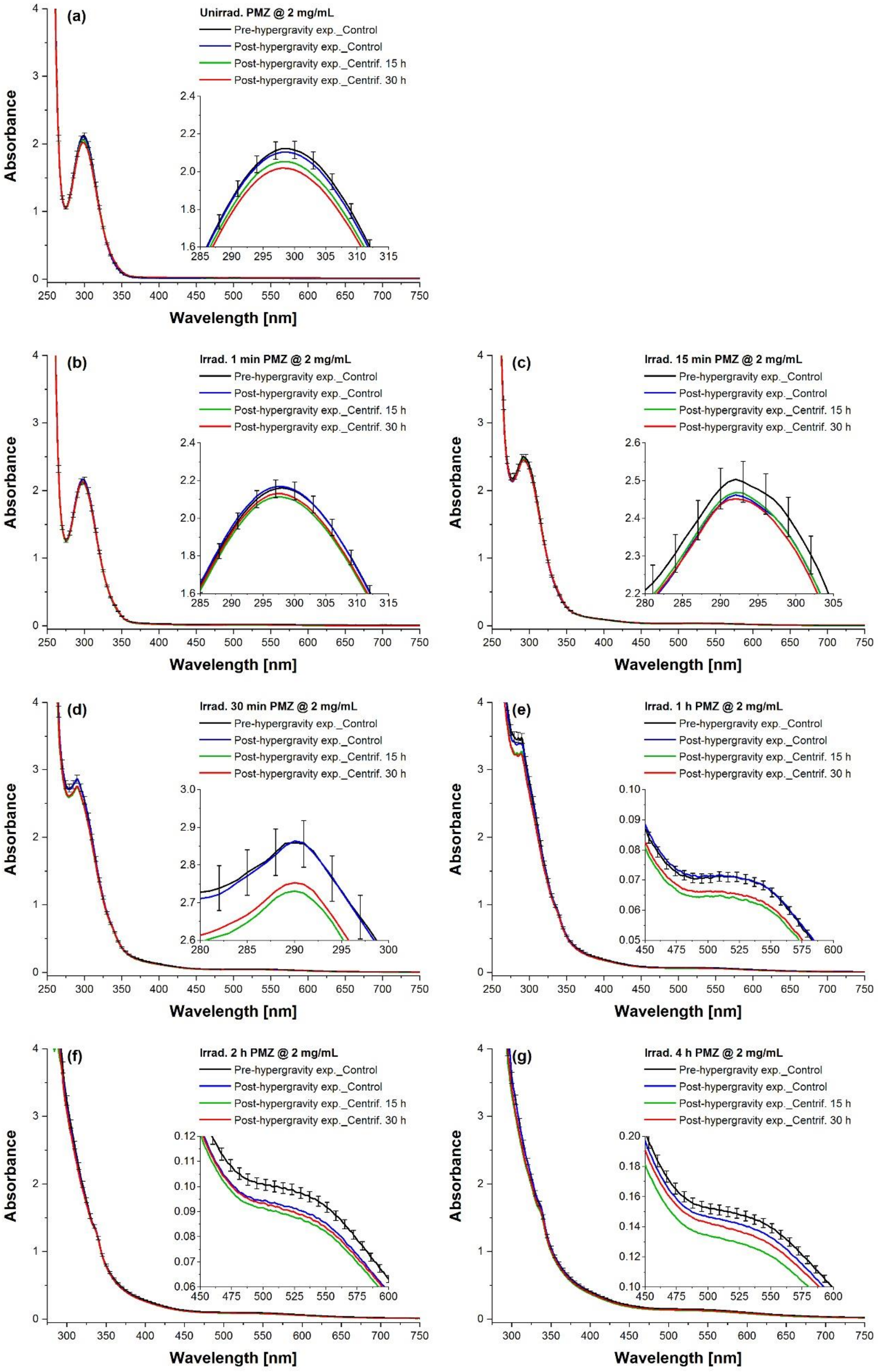

Figure S3. Spectra of (**a**) unirradiated, (**b**) 1 min, (**c**) 15 min (**d**) 30 min, (**e**) 1 h, (**f**) 2 h and (**g**) 4 h laser-irradiated 2 mg/mL PMZ uncentrifuged and centrifuged samples registered pre- and post-hypergravity exposure. Error bars: ± 2.174%.

Evolution of UV-Vis absorption spectra of 2 mg/mL TZ solutions subjected to terrestrial and hypergravity conditions

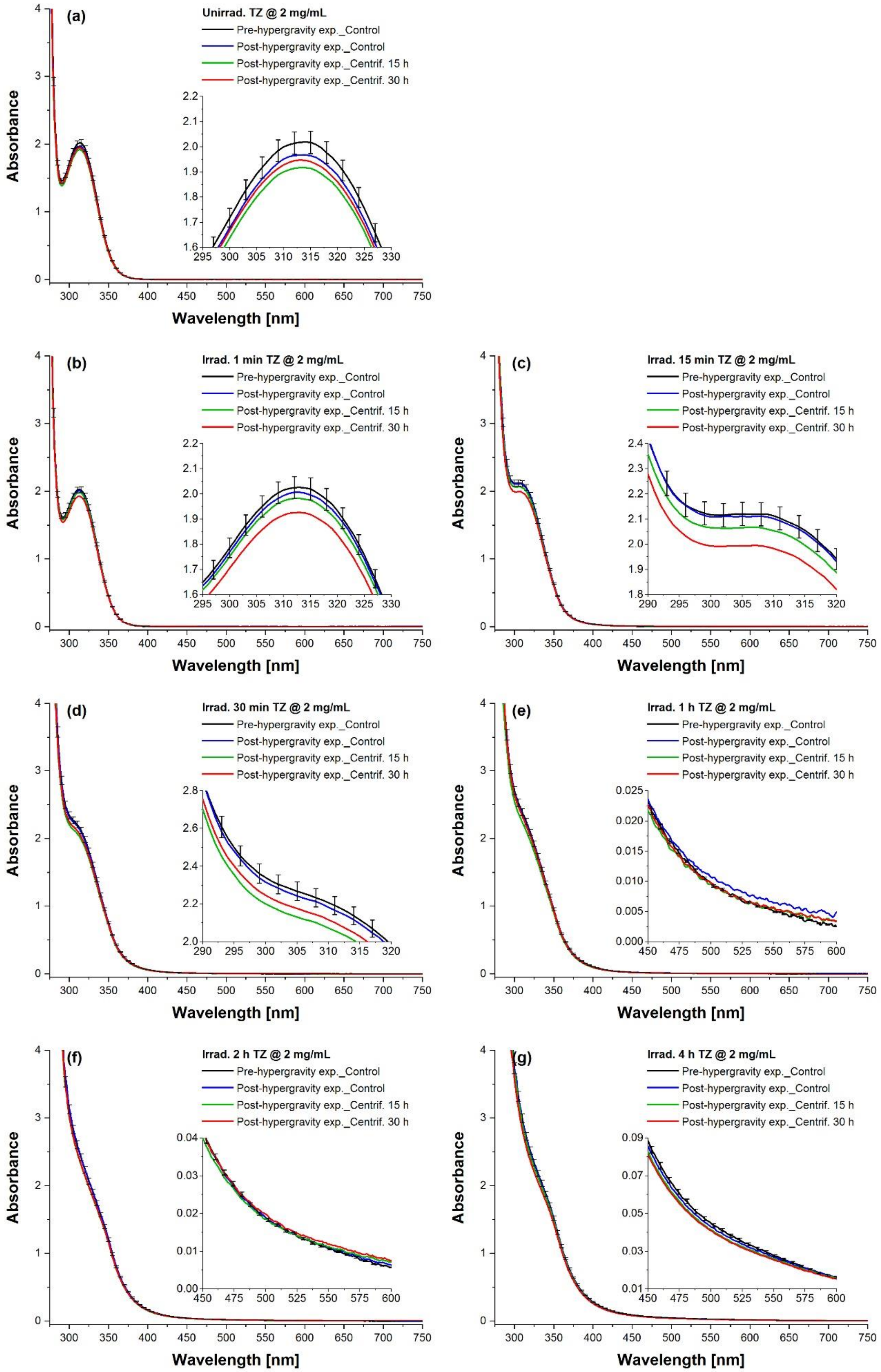

Figure S4. Spectra of (**a**) unexposed, (**b**) 1 min, (**c**) 15 min (**d**) 30 min, (**e**) 1 h, (**f**) 2 h and (**g**) 4 h laser-exposed 2 mg/mL TZ control and high-g treated samples recorded before and after the hypergravity experiment. Error bars: ± 2.174%.

Evolution of UV-Vis absorption spectra of 20 mg/mL PMZ solutions exposed to 1 and 20 g gravitational conditions

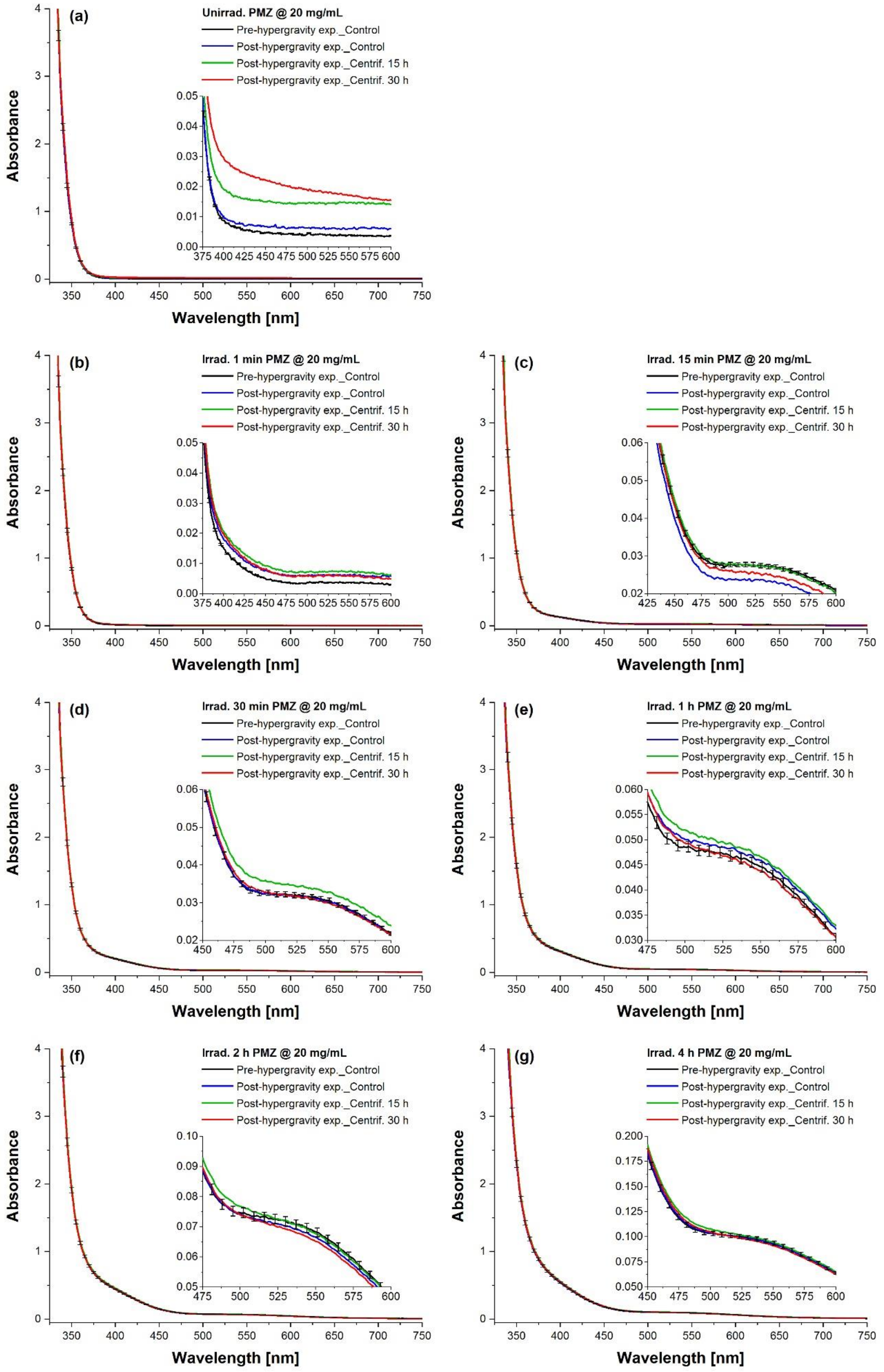

Figure S5. Spectra of (**a**) unirradiated, (**b**) 1 min, (**c**) 15 min (**d**) 30 min, (**e**) 1 h, (**f**) 2 h and (**g**) 4 h laser-irradiated 20 mg/mL PMZ control and high-g subjected samples registered pre- and post-centrifugation. Error bars: ± 2.174%.

Evolution of UV-Vis absorption spectra of 20 mg/mL TZ solutions subjected to terrestrial and hypergravity conditions

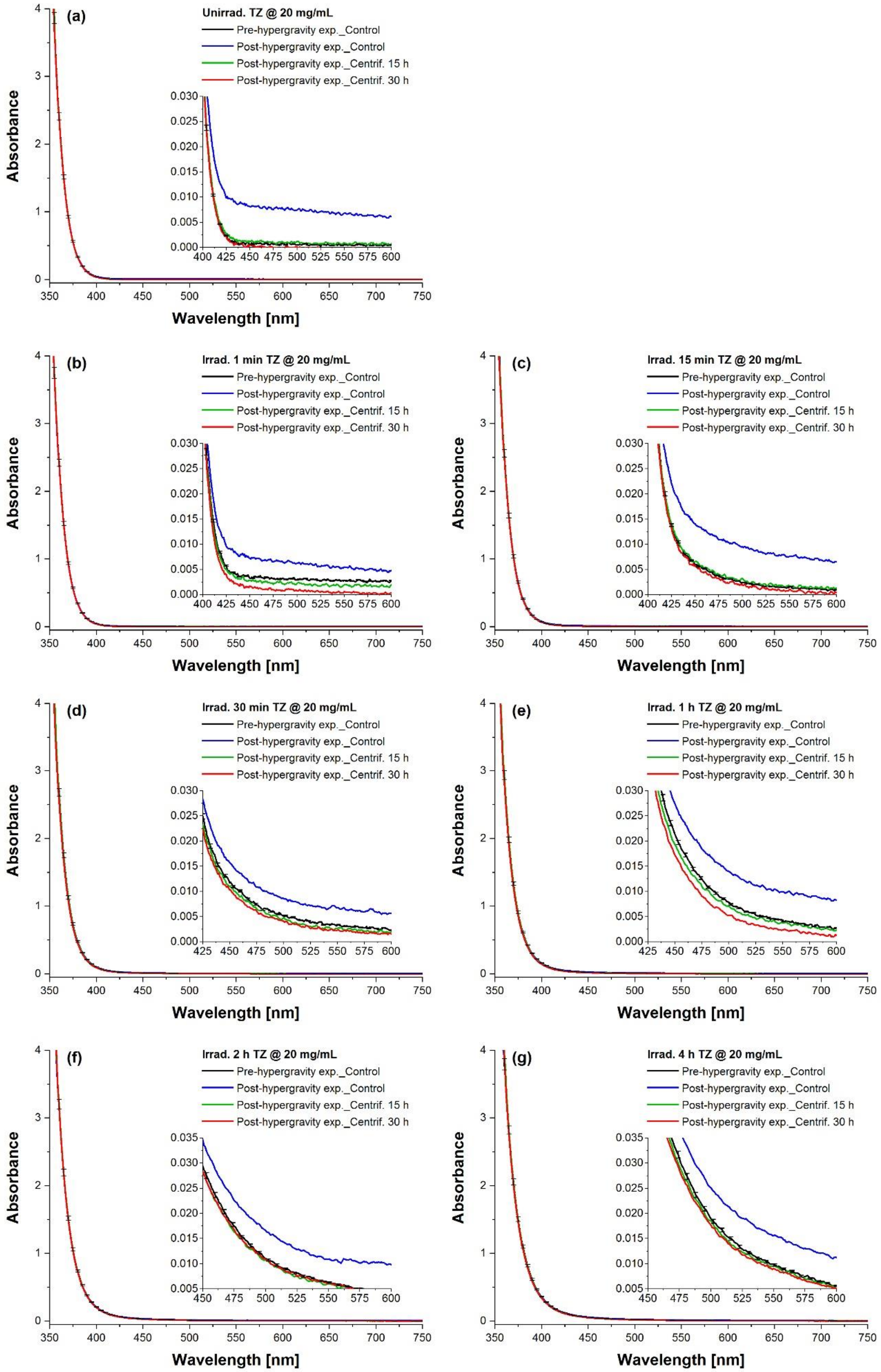

Figure S6. Spectra of (**a**) unexposed, (**b**) 1 min, (**c**) 15 min (**d**) 30 min, (**e**) 1 h, (**f**) 2 h and (**g**) 4 h laser-exposed 2 mg/mL TZ uncentrifuged and centrifuged samples recorded before and after the LDC experiment. Error bars: ± 2.174%.

Characteristic vibrational frequencies of the molecular bonds of PMZ

Table S2. Experimental and theoretical vibrational wavenumbers with their corresponding modes of vibrations obtained for unirradiated PMZ at 2 mg/mL by FTIR analysis before the hypergravity experiment.

Wavenumber [cm^{-1}]			Mode of vibration
Exp.	Theor. unscaled	Theor. scaled	
3055	3192	3066	asymmetric stretching vibration of =C−H of the ring system
3012	3117	2991	asymmetric stretching vibration of C−H from $-CH_3$
2980	3088	2967	asymmetric stretching vibration of C−H from $-CH_3$
2859	2973	2860	symmetric stretching vibration of C−H from $-CH_3$
1591	1637	1595	stretching vibration of the ring system
1569	1613	1573	stretching vibration of the ring system
1457	1487	1452	in-of-plane deformation vibration – scissoring of =C−H of the ring system<br>deformation vibrations – scissoring of C−H from $-CH_3$
1336	1360	1330	deformation vibrations – twisting of C−H from $-CH_3$<br>in-of-plane deformation vibration – rocking of =C−H of the ring system
1286	1314	1286	in-plane deformation vibration of the ring system<br>deformation vibrations – rocking of C−H from $-CH_3$
1256	1267	1240	in-of-plane deformation vibration – rocking of =C−H of the ring system<br>deformation vibrations – wagging of C−H from $-CH_3$
1227	1222	1199	stretching vibration of C−N (aromatic ring)<br>in-of-plane deformation vibration – rocking of =C−H of the ring system<br>deformation vibrations – wagging of C−H from $-CH_3$
1161	1187	1165	in-of-plane deformation vibration – scissoring of =C−H of the ring system
1128	1147	1126	in-of-plane deformation vibration – scissoring of =C−H of the ring system
1038	1058	1039	in-plane deformation vibration of the ring system
999	994	979	out-of-plane deformation vibration – wagging of =C−H of the ring system<br>deformation vibrations – wagging of C−H from $-CH_3$<br>deformation vibrations – twisting of C−H from $-CH_3$
934	942	929	out-of-plane deformation vibration – twisting of =C−H of the ring system
858	865	854	out-of-plane deformation vibration – twisting of =C−H of the ring system
754	765	756	out-of-plane deformation vibration – wagging of =C−H of the ring system

Characteristic vibrational frequencies of the molecular bonds of TZ

Table S3. Experimental and theoretical vibrational wavenumbers with their corresponding modes of vibrations identified pre- hypergravity within the FTIR spectra for unirradiated 2 mg/mL TZ.

Wavenumber [cm^{-1}]			Mode of vibration
**Exp.**	**Theor. unscaled**	**Theor. scaled**	
3054	3193	3064	=C−H stretching vibration
2954	3067	2949	=C−H stretching vibration
2866	2972	2861	C−H symmetric stretching vibration from CH_2
1560	1598	1559	stretching vibration of the aromatic ring C=C−C
1458	1486	1451	deformation vibration – scissoring of C−H from CH_2<br>stretching vibration of the aromatic ring C=C−C
1404	1429	1397	deformation vibration – wagging of C−H from pyridine ring<br>deformation vibrations – scissoring of C−H from −N−CH_3<br>in-plane deformation vibrations of =C−H (aromatic ring)
1317	1354	1324	in-plane deformation vibrations – wagging of C−H from S-CH_3<br>in-plane deformation vibrations of =C−H (aromatic ring)<br>deformation vibration – twisting of C−H from −$(CH_2)_2$ and pyridine ring
1279	1316	1287	in-plane deformation vibrations of =C−H (aromatic ring)<br>deformation vibration – wagging of C−H from −$(CH_2)_2$<br>deformation vibration – twisting of C−H from pyridine ring
1249	1271	1244	in-plane deformation vibrations of =C−H (aromatic ring)<br>deformation vibration – twisting of C−H from −$(CH_2)_2$ and pyridine ring
1131	1150	1128	in-plane deformation vibrations of =C−H (aromatic ring)
1110	1125	1104	in-plane deformation vibration of the ring system<br>deformation vibration – wagging of C−H from −N−CH_3<br>deformation vibration – twisting of C−H from pyridine ring
1038	1058	1040	=C−H in-plane deformation vibrations of 4-adjacent H atoms of the ring system<br>deformation vibration – wagging of C−H from pyridine ring<br>stretching vibration of the aromatic ring C=C−C
968	994	978	in-plane deformation vibrations – wagging of C−H from S−CH_3
930	947	931	in-plane deformation vibration of the ring system<br>deformation vibration – twisting of C−H from −$(CH_2)_2$, −N−CH_3 and pyridine ring
906	916	904	in-plane deformation vibration of the ring system<br>deformation vibration – twisting of C−H from −$(CH_2)_2$ and pyridine
848	857	845	deformation vibration – rocking of C−H from −CH_2<br>deformation vibration – wagging of C−H from −N−CH_3
804	824	814	out-of-plane deformation vibration of the ring system<br>deformation vibration – wagging of C−H from S−CH_3
751	768	760	deformation vibration – rocking of C−H from −$(CH_2)_2$<br>out-of-plane deformation vibration of the ring system

Displacement vectors (blue arrows) illustrating the motion associated with the IR vibrations for the representative wavenumbers from unirradiated PMZ IR spectra

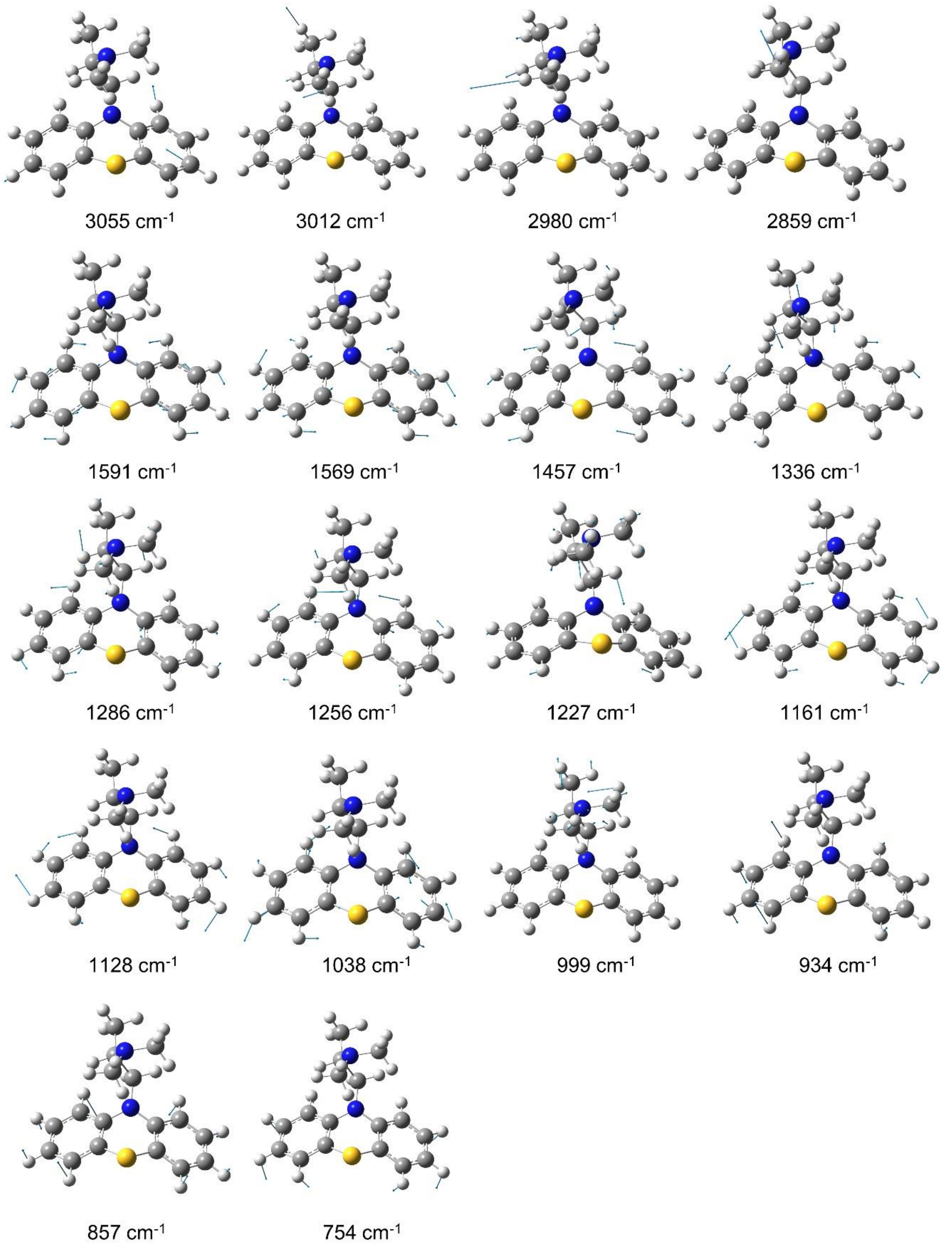

Figure S7. Annotation: white – H, grey – C, blue – N and yellow – O.

Displacement vectors (blue arrows) showing the motion associated with the IR vibrations for the representative wavenumbers from unirradiated TZ IR spectra

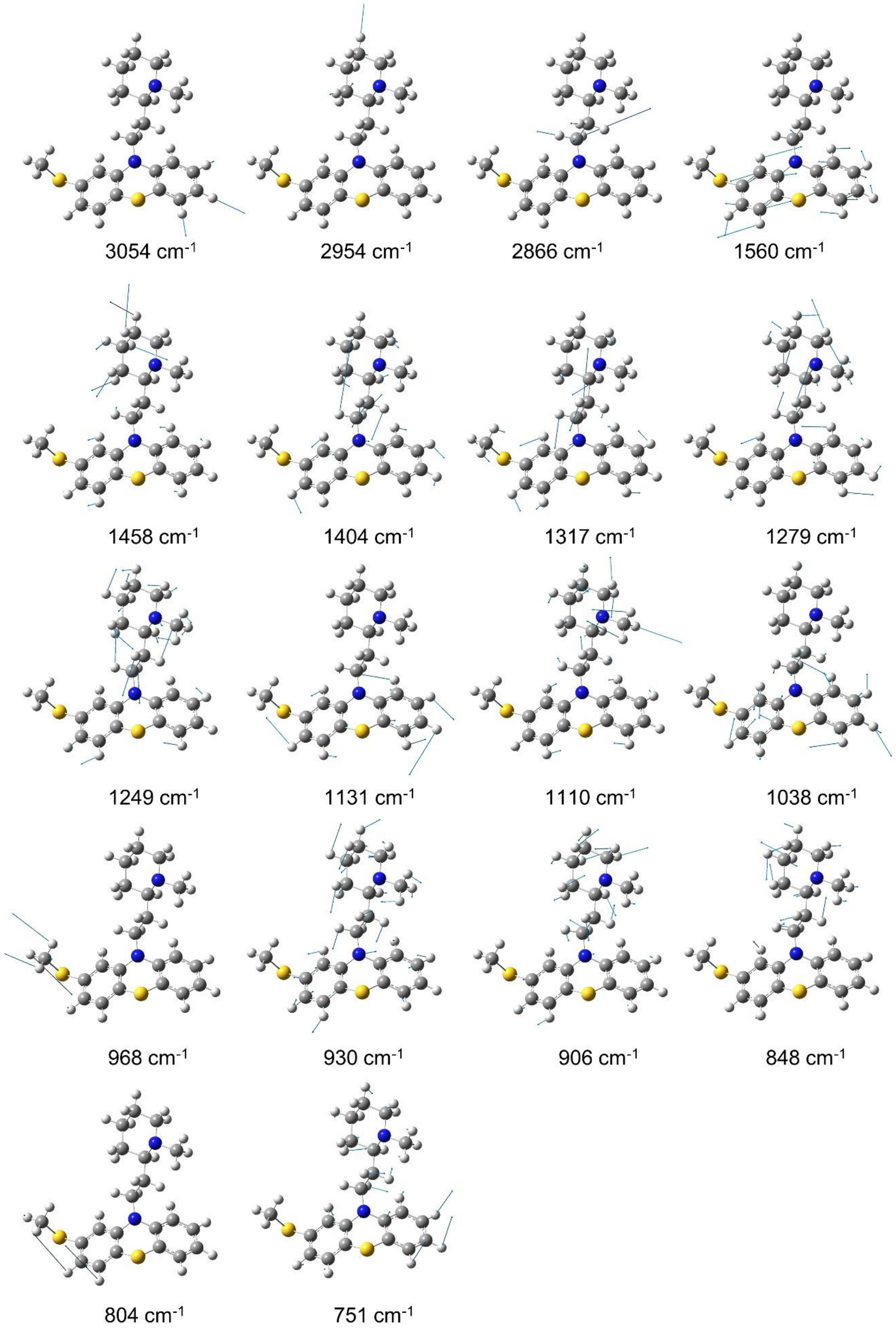

Figure S8. Annotation: white – H, grey – C, blue – N and yellow – O.

Evolution of FTIR absorption spectra of 2 mg/mL PMZ solutions pre- and post-hypergravity

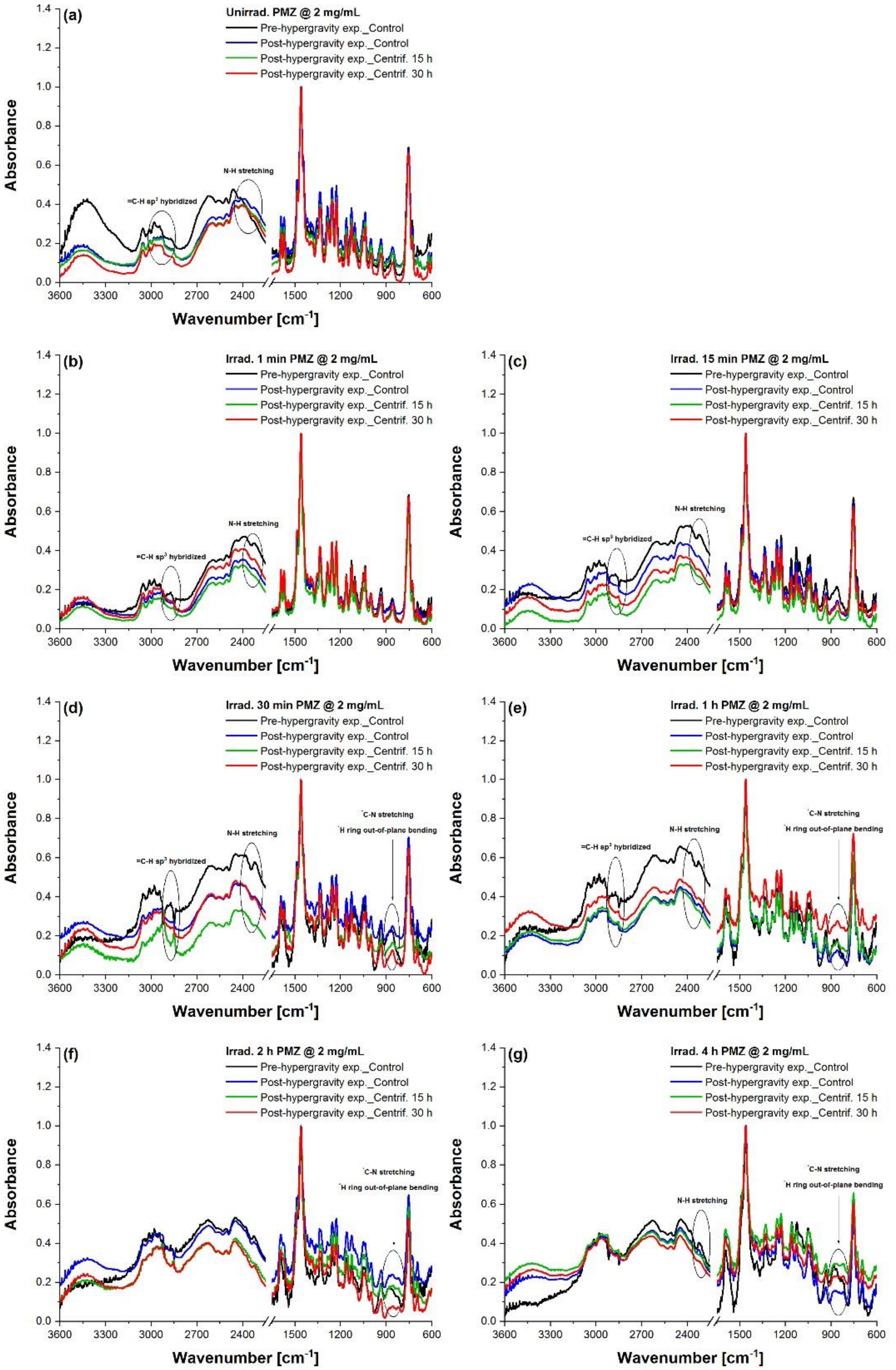

Figure S9. Spectra of (**a**) unirradiated, (**b**) 1 min, (**c**) 15 min (**d**) 30 min, (**e**) 1 h, (**f**) 2 h and (**g**) 4 h laser-irradiated 2 mg/mL PMZ control and high-g treated samples recorded before and after centrifugation.

Evolution of FTIR absorption spectra of 2 mg/mL TZ solutions subjected to 1 and 20 g conditions

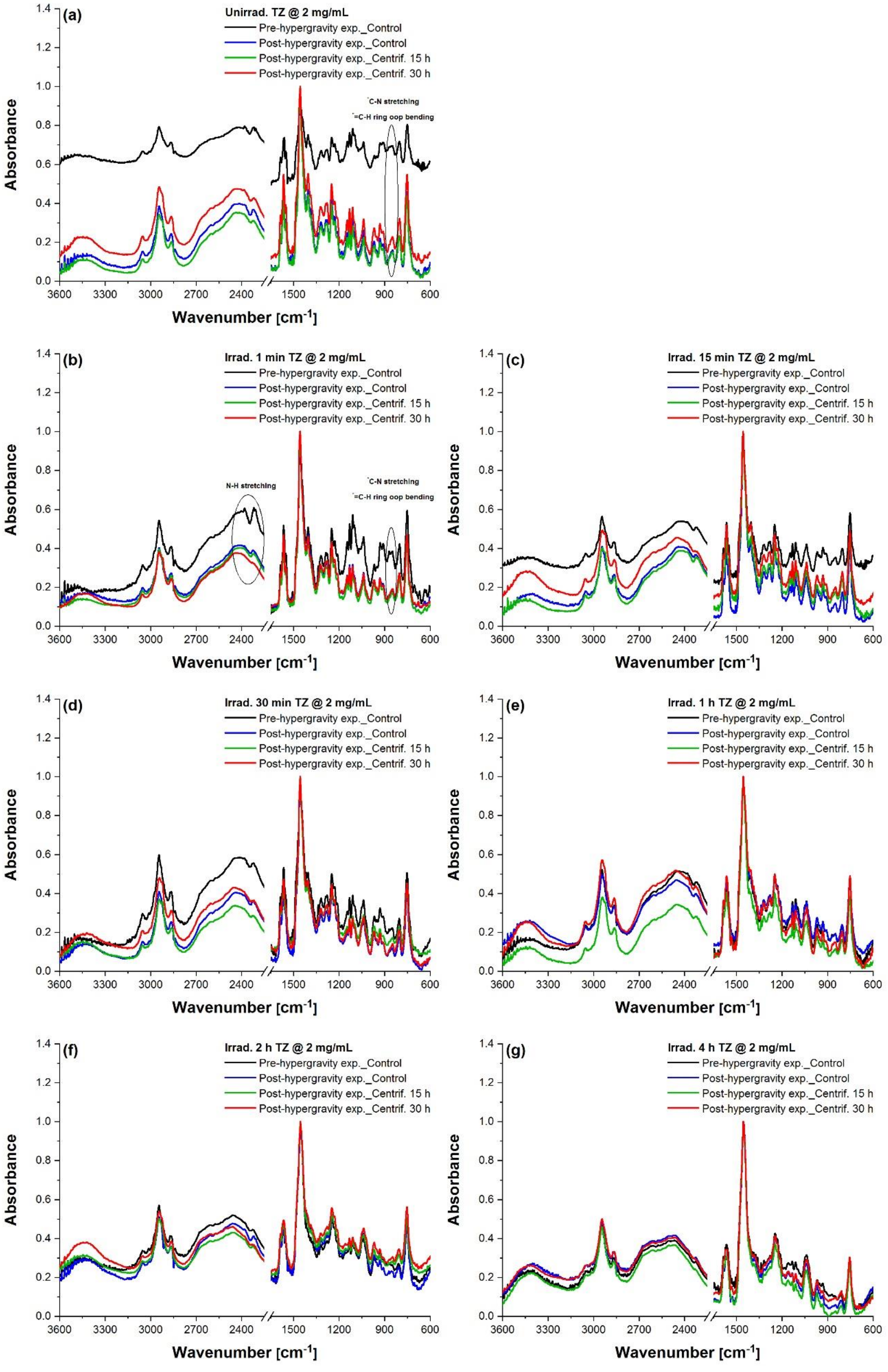

Figure S10. Spectra of (**a**) unexposed, (**b**) 1 min, (**c**) 15 min (**d**) 30 min, (**e**) 1 h, (**f**) 2 h and (**g**) 4 h laser-exposed 2 mg/mL TZ uncentrifuged and centrifuged samples registered before and after the hypergravity experiment.

Evolution of FTIR absorption spectra of 20 mg/mL PMZ solutions exposed to terrestrial and hypergravity conditions

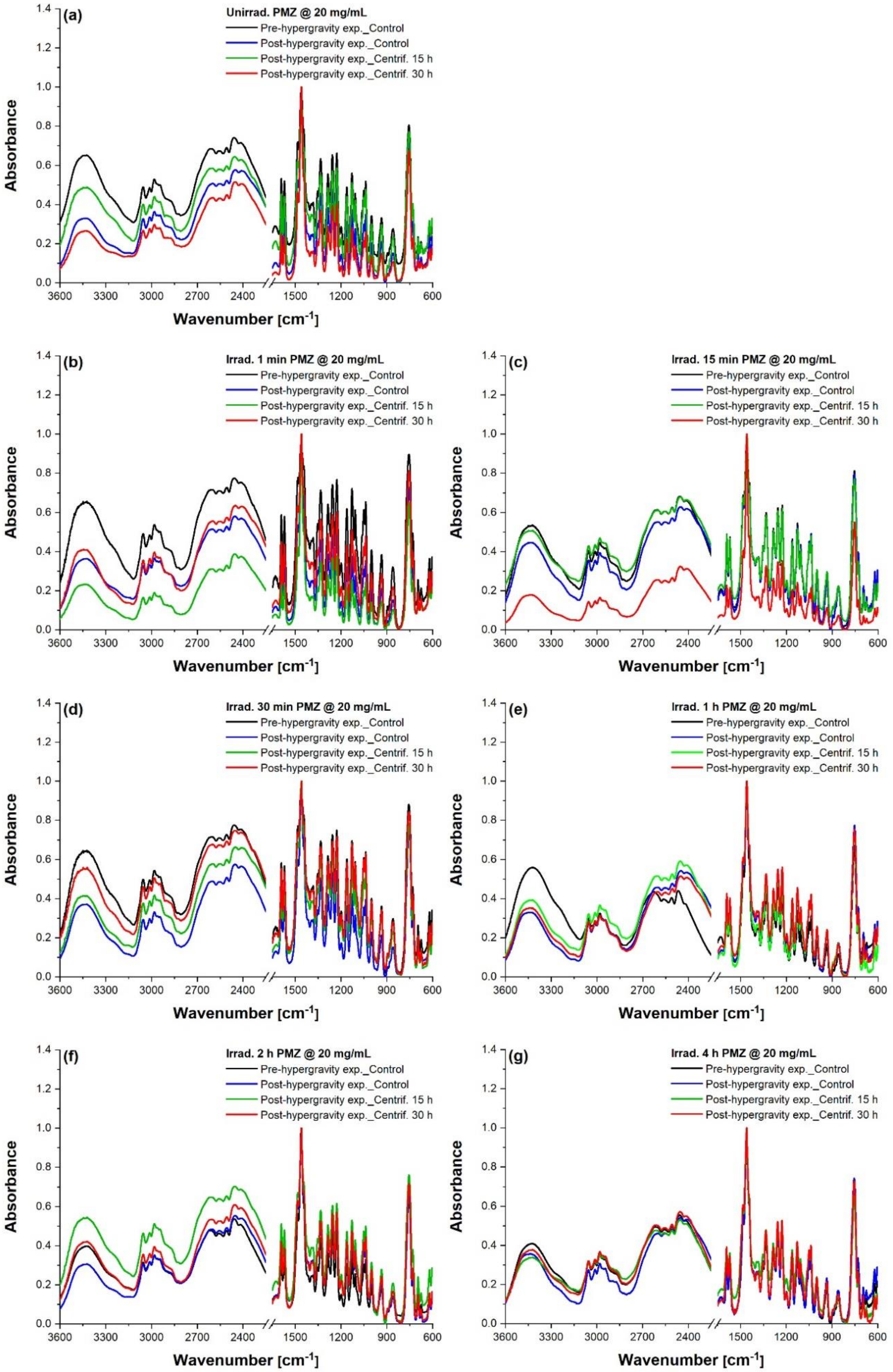

Figure S11. Spectra of (**a**) unirradiated, (**b**) 1 min, (**c**) 15 min (**d**) 30 min, (**e**) 1 h, (**f**) 2 h and (**g**) 4 h laser-irradiated 20 mg/mL PMZ control and high-g treated samples recorded pre- and post-centrifugation.

Evolution of FTIR absorption spectra of 20 mg/mL TZ solutions subjected to 1 and 20 g conditions

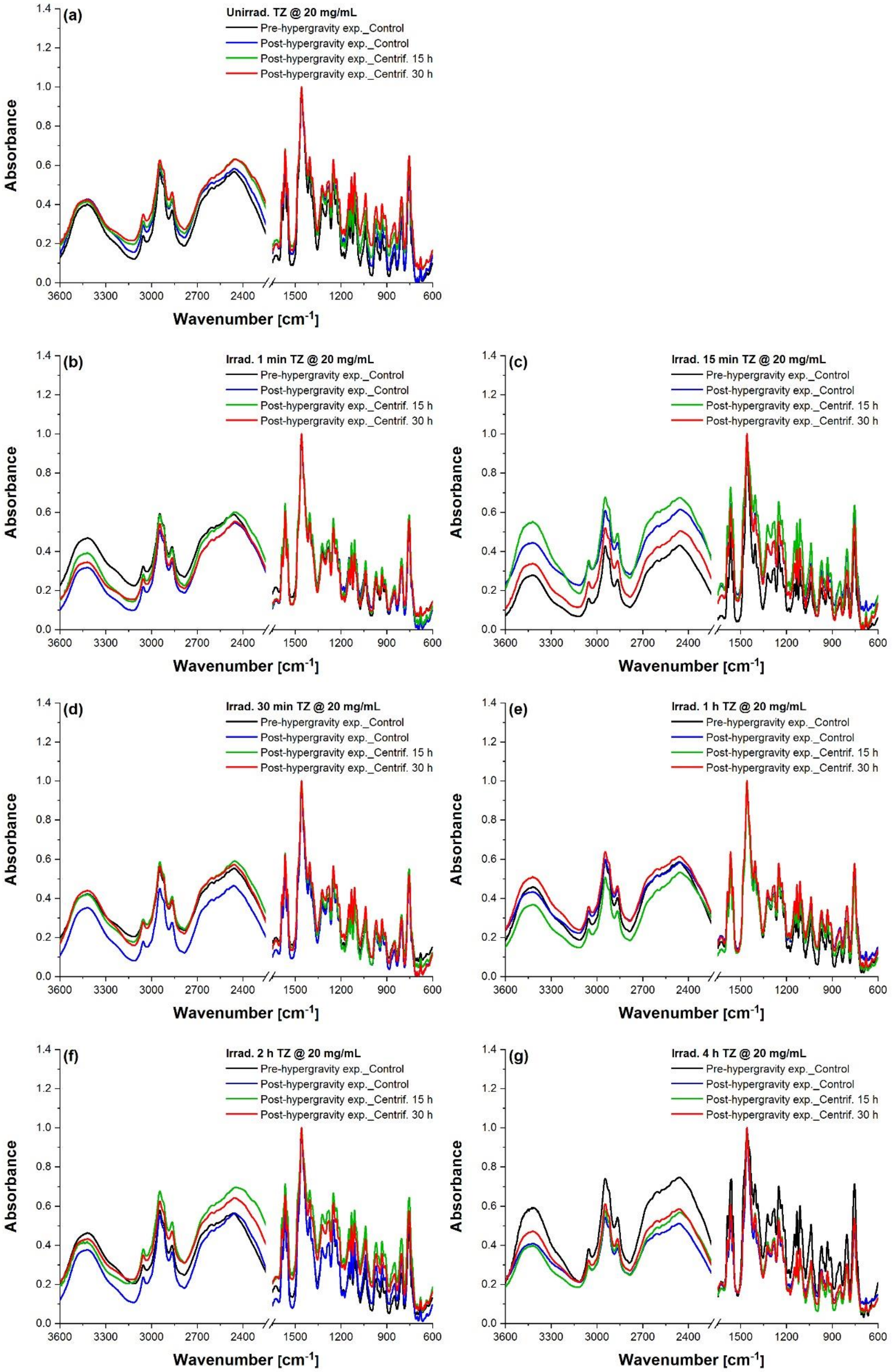

Figure S12. Spectra of (**a**) unexposed, (**b**) 1 min, (**c**) 15 min (**d**) 30 min, (**e**) 1 h, (**f**) 2 h and (**g**) 4 h laser-exposed 20 mg/mL TZ uncentrifuged and centrifuged samples registered before and after the LDC experiment.
